# MSFusion: Multi-Scale Cross-Modal Fusion with Adaptive Attention for Multimodal Medical Image Fusion

**DOI:** 10.3390/bios16080423

**Published:** 2026-08-06

**Authors:** Liu Wang, Yang Zhou, Wenjia Li, Lijuan Shi

**Affiliations:** 1 School of Computer Science and Technology, Changchun University, 6543 Weixing Road, Changchun 130022, China; wangl95@ccu.edu.cn (L.W.); 241501497@mails.ccu.edu.cn (Y.Z.); 241503553@mails.ccu.edu.cn (W.L.); 2 Jilin Provincial Key Laboratory of Human Health Status Identification Function & Enhancement, 6543 Weixing Road, Changchun 130022, China

**Keywords:** multimodal medical image fusion, multi-scale cross-modal cooperative fusion, multi-scale adaptive fusion, Vision RWKV

## Abstract

Multimodal medical image fusion integrates complementary information from heterogeneous imaging modalities to provide comprehensive visual support for clinical analysis. Most existing methods adopt an “encode–fuse–decode” paradigm that applies a single fusion rule only at the deepest network layer, often discarding shallow detail features and yielding blurred outputs with poor textural fidelity. To address this limitation, we propose MSFusion, a novel hierarchical framework that distributes adaptive fusion throughout the entire decoder stage. By leveraging skip connections to align decoder layers with corresponding encoder features, MSFusion enables full-scale integration of multi-resolution representations. The architecture employs a dual-branch convolutional encoder and introduces two core modules in the decoder: (1) the Multi-Scale Adaptive Fusion (MSAF) module, which addresses insufficient exploitation of cross-scale complementarity by dynamically weighting features via learnable attention, thereby balancing fine details and global semantics, and (2) the Multi-Scale Cross-Modal Cooperative Fusion (MSCMCF) module, which mitigates semantic misalignment through a cross-modal interactive attention mechanism that establishes robust inter-modality correspondences and promotes deep feature alignment. Additionally, a Vision RWKV (VRWKV) block is integrated to efficiently model both local and global spatial dependencies with linear computational complexity. Extensive experiments on public CT–MRI, PET–MRI, and SPECT–MRI datasets are evaluated using six standard metrics. On the CT–MRI benchmark, our method achieves MSE (↓) = 0.0423, CC = 0.8198, and SCD = 0.7023, outperforming state-of-the-art approaches. These results—combined with superior visual quality—demonstrate that MSFusion sets a new standard for accurate, detailed, and clinically meaningful multimodal image fusion.

## 1. Introduction

Due to the inherent limitations of single-modality imaging in simultaneously capturing both structural and functional information, multimodal fusion offers a powerful strategy to overcome these complementary deficits [1]. Specifically, anatomical modalities—such as computed tomography (CT) and magnetic resonance imaging (MRI)—excel at delineating tissue morphology, organ boundaries, and spatial relationships, thereby providing a robust anatomical scaffold for lesion localization. In contrast, functional modalities—including positron emission tomography (PET) and single-photon emission computed tomography (SPECT)—are highly sensitive to physiological processes such as metabolic activity and perfusion, enabling early detection and pathological characterization of abnormalities [2]. By synergistically integrating structural fidelity with functional insight, multimodal fusion generates composite images that preserve fine anatomical detail while enriching diagnostic content, empowering clinicians to concurrently assess both the spatial location and biological behavior of lesions [3,4,5]. Motivated by this clinical imperative, this work aims to develop a high-fidelity fusion framework that faithfully preserves and harmonizes complementary information across modalities.

Early approaches to medical image fusion (MIF) were predominantly rule-based, structured around a three-stage pipeline: decomposition, fusion, and reconstruction. These methods relied on handcrafted fusion rules, which can be broadly categorized into three paradigms. The earliest—spatial-domain direct fusion [6,7,8]—operated on pixel-level saliency or local energy measures within fixed windows. While computationally efficient, these strategies ignored global semantic coherence, often yielding fused images plagued by structural blurring and loss of fine detail. To address this, multi-scale transform-based methods [9,10,11] emerged, leveraging Laplacian pyramids, discrete wavelet transforms, or non-subsampled contourlet transforms (NSCT) to decompose images into low- and high-frequency sub-bands. This enabled simultaneous preservation of global anatomy and local texture, yet still employed static fusion rules incapable of adapting to the intrinsic heterogeneity between modalities. Subsequently, sparse representation and dictionary-learning-based approaches [12,13,14] advanced the field by encoding image patches over overcomplete dictionaries, thereby enhancing cross-modal correlation and robustness to noise. However, these methods suffered from prohibitive computational costs, sensitivity to hyperparameter tuning, and limited generalizability across diverse clinical datasets—highlighting a persistent gap between theoretical expressiveness and practical deployability.

In recent years, deep learning-based approaches have demonstrated remarkable success in medical image fusion by leveraging data-driven feature extraction and adaptive fusion strategies—surpassing the rigid, handcrafted rules of classical methods. Convolutional neural networks (CNNs), in particular, have become a cornerstone due to their efficacy in capturing local spatial patterns, as exemplified by frameworks such as FRAC-HCM [8] and GeSeNet [15]. However, the inherently local receptive fields of CNNs limit their capacity to model long-range dependencies; even with stacked convolutions and pooling operations, global contextual information remains incompletely and inefficiently encoded [16,17]. This limitation has spurred the adoption of Vision Transformers, which exploit self-attention mechanisms to establish holistic pixel-to-pixel relationships across the entire image, thereby preserving global semantics—as seen in Bsafusion [18], MM-Net [19], and EMMA [20]. Yet, neither architecture alone is sufficient: CNNs underrepresent global structure, while pure Transformers often neglect fine-grained local textures and incur high computational overhead. Consequently, hybrid CNN–Transformer architectures have emerged, typically cast within an encoder–decoder framework to harness the strengths of both paradigms. Nevertheless, this design frequently suffers from incomplete fusion of complementary modality-specific information, as critical features may be lost or diluted during encoding or upsampling. While some studies attempt to mitigate this through modular decomposition [21] or auxiliary reconstruction losses via autoencoders [22], these remedies remain palliative—they do not guarantee lossless, hierarchical information flow from input to output.

Recent deep learning-based medical image fusion methods can be further categorized according to the location and organization of cross-modal fusion. First, centralized latent feature fusion methods, exemplified by M4FNet [22] and MACTFusion [23], generally extract modality-specific deep representations, perform attention- or Transformer-based interaction in a dedicated fusion stage, and subsequently reconstruct the fused image. Second, Transformer-enhanced approaches, including MATR [24], SwinFusion [25], and FATFusion [26], CrossFuse [27] improve global dependency modeling and cross-modal communication through multi-scale self-attention, cross-attention, or modality-guided Transformer modules. Nevertheless, their cross-modal interactions are still organized around a limited set of designated fusion blocks between feature extraction and image reconstruction. Third, more recent multi-scale or decoder-aware approaches, such as the dual-scale weighted residual attention network [28] and MARCFusion [29], introduce feature aggregation at multiple resolutions or within a nested decoder. These approaches narrow the gap between centralized and hierarchical fusion; however, they do not establish a unified decoder-wide mechanism in which bidirectional cross-modal alignment and input-conditional scale balancing are jointly and repeatedly performed at every reconstruction resolution.

In contrast, MSFusion reformulates fusion as a distributed and progressive reconstruction process. At each decoder level, resolution-matched features from the two modality-specific encoders are reintroduced through skip connections and processed by the MSCMCF and MSAF modules, followed by VRWKV-based global spatial modeling. Consequently, fusion is no longer treated as a one-off or sparsely placed latent space operation; instead, it is distributed throughout the entire decoder, allowing shallow textural details and deep semantic information to be progressively aligned, adaptively weighted, and integrated. Accordingly, the central research question addressed in this work is how can cross-modal fusion be distributed throughout the decoding pathway so that modality alignment, multi-scale information selection, and efficient global context modeling are jointly achieved at every resolution.

The main contributions of this work are summarized as follows:

• To address the shallow detail loss in centralized fusion, we propose MSFusion, a hierarchical progressive framework that distributes adaptive fusion across all decoder stages, ensuring the lossless integration of fine-grained textures and deep semantic representations.
• To overcome modality dominance in unidirectional cross-attention, we develop the Multi-Scale Cross-Modal Cooperative Fusion (MSCMCF) module. Its strictly bidirectional attention mechanism guarantees symmetric information exchange, ensuring robust semantic alignment between modalities.
• To resolve the rigidity of static fusion rules, we design the Multi-Scale Adaptive Fusion (MSAF) module, which employs an input-conditional weight prediction network to dynamically balance global semantics and local details at each scale.
• To eliminate the prohibitive O(T^2^) computational bottleneck of standard Vision Transformers in high-resolution medical imaging, we introduce the Vision RWKV (VRWKV) block, achieving exact O(T) linear complexity for global spatial modeling without any approximation errors.

## 2. Related Work

### 2.1. Traditional Methods

Over the past few decades, the majority of multimodal medical image fusion (MMIF) approaches have relied on traditional signal processing techniques. The field was initially shaped using pyramid-based methods, notably the Laplacian pyramid fusion algorithm introduced in [12]. This was followed by a wave of multi-scale decomposition (MSD)-based strategies, which sought to better preserve structural details across spatial frequencies. Representative works include a phase congruency- and local Laplacian energy-driven fusion method for multimodal medical images [30]; an approach based on non-subsampled contourlet transform (NSCT) [31]; a Laplacian pyramid-based multi-scale fusion framework tailored for medical imaging [32]; a discrete wavelet transform (DWT)-based technique for multimodal fusion [33]; and a multi-focus image fusion method combining stationary wavelet transform with non-local means filtering [34]. Subsequently, sparse representation emerged as a powerful alternative, offering data-adaptive basis learning. Pioneering work by [35] first introduced sparse coding into image fusion, proposing a sparse representation-based method for multi-focus imagery. This paradigm quickly gained traction, leading to a series of advances: an adaptive sparse representation (ASR) model [36], a convolutional sparse decomposition (CSD)-based fusion scheme [37], a novel sparse coding strategy for multi-focus fusion [38], and a hybrid framework integrating image decomposition with sparse representation for multimodal medical image fusion [13]. Despite their successes, these traditional methods are fundamentally constrained by handcrafted features and fixed fusion rules, which lack the capacity to adapt to the complex, heterogeneous nature of cross-modal medical data. These limitations have motivated a shift toward learning-based paradigms capable of end-to-end optimization and semantic-aware fusion—a transition that underpins the motivation for our work.

### 2.2. Deep Learning-Based Methods

With the rapid advancement of deep learning, data-driven approaches have gradually supplanted traditional handcrafted methods as the dominant paradigm in multimodal medical image fusion. Early efforts centered on convolutional neural networks (CNNs), leveraging their strong local feature extraction capabilities; for instance, ref. [39] proposed a general-purpose CNN-based fusion framework, while [22] introduced a method that used CNNs to jointly learn feature representation and fusion rules. However, the inherently local receptive fields of CNNs limited their ability to capture long-range dependencies, prompting the integration of Vision Transformers, which excel at global context modeling. This shift led to several hybrid architectures: ref. [40] developed a dual-branch Transformer–CNN fusion framework; ref. [23] designed an edge-enhanced fusion model incorporating a Sobel operator to extract edge priors and a cross-scale Transformer module that captures multi-scale global information through hierarchical embeddings, employing a multi-path strategy to decouple shallow and deep features and optimizing fusion quality via perceptual and gradient losses; ref. [41] proposed BTMF-GAN, featuring a multi-Beyond the medical image fusion domain, recent advances in efficient Transformer design and mutual guidance paradigms offer valuable insights. Liu et al. [42] proposed a Low-Rank Transformer Network (LRTN) for hyperspectral computational imaging that leverages spatial low-rank matrix factorization to reduce the quadratic complexity of self-attention while maintaining fusion quality, sharing conceptual motivation with our VRWKV module’s linear complexity design, albeit in a different imaging modality. Hong et al. [43] introduced MERF, a mutual-guided learning framework for multi-exposure image fusion that jointly optimizes registration and fusion through a dual attention network, demonstrating the effectiveness of cross-modal mutual guidance strategies beyond the medical domain. These works further validate the broader trend toward efficient attention mechanisms and mutual-guidance paradigms in image fusion, reinforcing the design principles underlying MSFusion.

More recently, a diverse array of deep learning paradigms has been explored to further advance multimodal medical image fusion. Transfer learning has emerged as a practical strategy to mitigate data scarcity in medical imaging: Liang [44] proposed a deep medical image fusion (DMIF) framework that couples low-rank representation (LatLRR) decomposition with pre-trained VGG-16 features, decomposing source images into principal and salient components and fusing them through a weighted-average rule, achieving consistent improvements across CT–MRI, MRI–PET, MRI–SPECT, and MR-T1/MR-T2 benchmarks. Similarly, Do et al. [45] combined a modified VGG19 for high-frequency feature extraction with an equilibrium optimization algorithm (EOA) for adaptive low-frequency fusion, demonstrating that optimization-guided transfer learning can outperform seven state-of-the-art methods across multiple modality pairs. In parallel, attention mechanisms have been widely adopted to enhance feature selectivity in fusion architectures: Poornima and Anand [28] designed a dual-scale weighted fusion residual attention network with an encoder–decoder architecture, where residual attention blocks refine multi-scale features before weighted aggregation at dual resolutions, yielding improved structural fidelity for CT–MRI fusion. Addressing the challenge of cross-modal semantic alignment, Tang et al. [29] proposed MARCFusion, an adaptive residual cross-domain fusion network that jointly leverages spatial-domain and frequency-domain fusion modules—specifically, residual spatial-domain fusion blocks and residual frequency-domain fusion blocks—within a nested connection decoder, trained with a composite detail-and-saliency loss in an unsupervised manner. Beyond the image fusion domain, the hierarchical multi-scale feature fusion paradigm has proven broadly effective: Huo et al. [46] introduced HiFuse [47], a three-branch hierarchical network that fuses global and local features through an adaptive hierarchical feature fusion (HFF) block for medical image classification, demonstrating that multi-scale fusion strategies generalize across diverse medical imaging tasks. Despite these advances, a common limitation persists: most existing methods either apply fusion at a single network depth, rely on unidirectional attention, or lack explicit mechanisms for bidirectional cross-modal alignment across multiple scales, gaps that the proposed MSFusion framework, with its decoder-stage Multi-Scale Adaptive Fusion (MSAF) and cross-modal cooperative fusion (MSCMCF) modules, is specifically designed to address.

## 3. Methods

### 3.1. Overall Architecture

**Figure 1:**
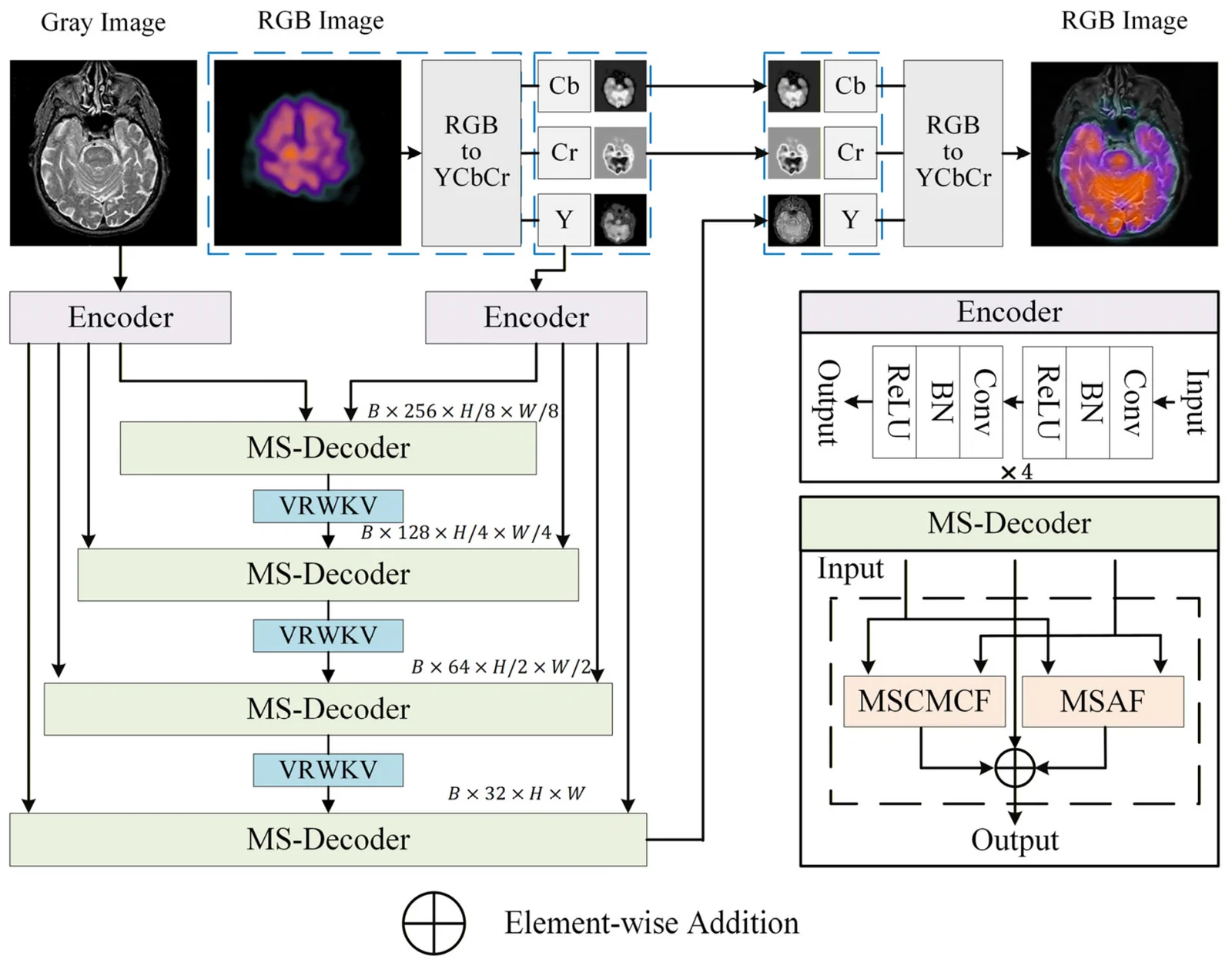
Overall architecture of the proposed MSFusion framework. The framework employs a dual-branch encoder to hierarchically extract multi-scale features from two co-registered source modalities (e.g., CT and MRI). These features are progressively fused in the MS-Decoder stages through the Multi-Scale Cross-Modal Cooperative Fusion (MSCMCF) and Multi-Scale Adaptive Fusion (MSAF) modules, followed by global spatial modeling via the Vision RWKV (VRWKV) block. Skip connections are utilized to preserve shallow textural details, ultimately reconstructing a high-fidelity, diagnostically enriched fused image.

The proposed MSFusion framework is illustrated in Figure 1. Given two co-registered multimodal medical images $I_{A}\in R^{H\times W\times C}$ and $I_{B}\in R^{H\times W\times C}$, we introduce an end-to-end deep network designed to generate a fused image $I_{F}\in R^{H\times W\times C}$ that synergistically integrates complementary structural and functional information, thereby enhancing diagnostic accuracy, lesion localization, and clinical decision support. The architecture follows an encoder–decoder paradigm composed of two symmetric parallel encoder branches, a cascade of multi-level MS-Decoder modules, and Vision RWKV (VRWKV) spatial enhancement units. Each input modality is independently processed through a dedicated encoder branch consisting of four sequentially stacked convolutional blocks. The output channel dimensions of the four blocks are set to [32, 64, 128, 256], respectively. Each block contains two 3×3 convolutional layers, and each convolution is followed by batch normalization and a ReLU activation. In the first block, both convolutional layers use a stride of 1, thereby preserving the original spatial resolution. In each of the subsequent three blocks, the first convolutional layer uses a stride of 2 for spatial downsampling, whereas the second convolutional layer uses a stride of 1. A padding of 1 is used for all convolutional layers. Consequently, given an input tensor of size B×1×H×W, the four blocks generate hierarchical feature maps of sizes B×32×H×W, B×64×H/2×W/2, B×128×H/4×W/4, and B×256×H/8×W/8, respectively. These multi-resolution features are transferred to the corresponding decoder stages through skip connections for progressive cross-modal fusion. At every resolution level, the corresponding feature maps from both encoders are fed into an MS-Decoder: the first MS-Decoder receives the deepest encoder outputs directly, while subsequent MS-Decoders take as input both the output from the previous decoder stage and the skip-connected features from the corresponding encoder levels. Within each MS-Decoder, two novel components operate in tandem: (1) the Multi-Scale Cross-Modal Cooperative Fusion (MSCMCF) module, which establishes deep semantic correspondence between modalities, enhances complementary features, and suppresses modality-specific noise, and (2) the Multi-Scale Adaptive Fusion (MSAF) module, which dynamically assigns fusion weights across scales to optimally balance global semantics and local details. The outputs of these two blocks are combined via element-wise addition. Following each MS-Decoder, a VRWKV module is inserted to strengthen long-range spatial interactions and improve feature consistency through RWKV-based global context modeling. Through progressive upsampling and repeated integration of low-level details via skip connections—also fused by element-wise addition—the decoder reconstructs a high-fidelity, diagnostically enriched fusion image $I_{F}$ that preserves both anatomical precision and functional relevance. For clarity, a comprehensive summary of all mathematical symbols, abbreviations, and module-specific terminology used throughout the manuscript is provided in Supplementary Table S1.

Information flow through one decoder stage: Data flows through the fixed sequence MSCMCF → MSAF → VRWKV. First, MSCMCF receives aligned bimodal features and outputs cross-modally enhanced features. Second, MSAF takes these features and produces scale-adaptively fused features by dynamically weighting global semantics and local details. Third, VRWKV processes the fused features to model long-range dependencies. This sequence recurs across four resolution levels.

### 3.2. Multi-Scale Cross-Modal Cooperative Fusion

**Figure 2:**
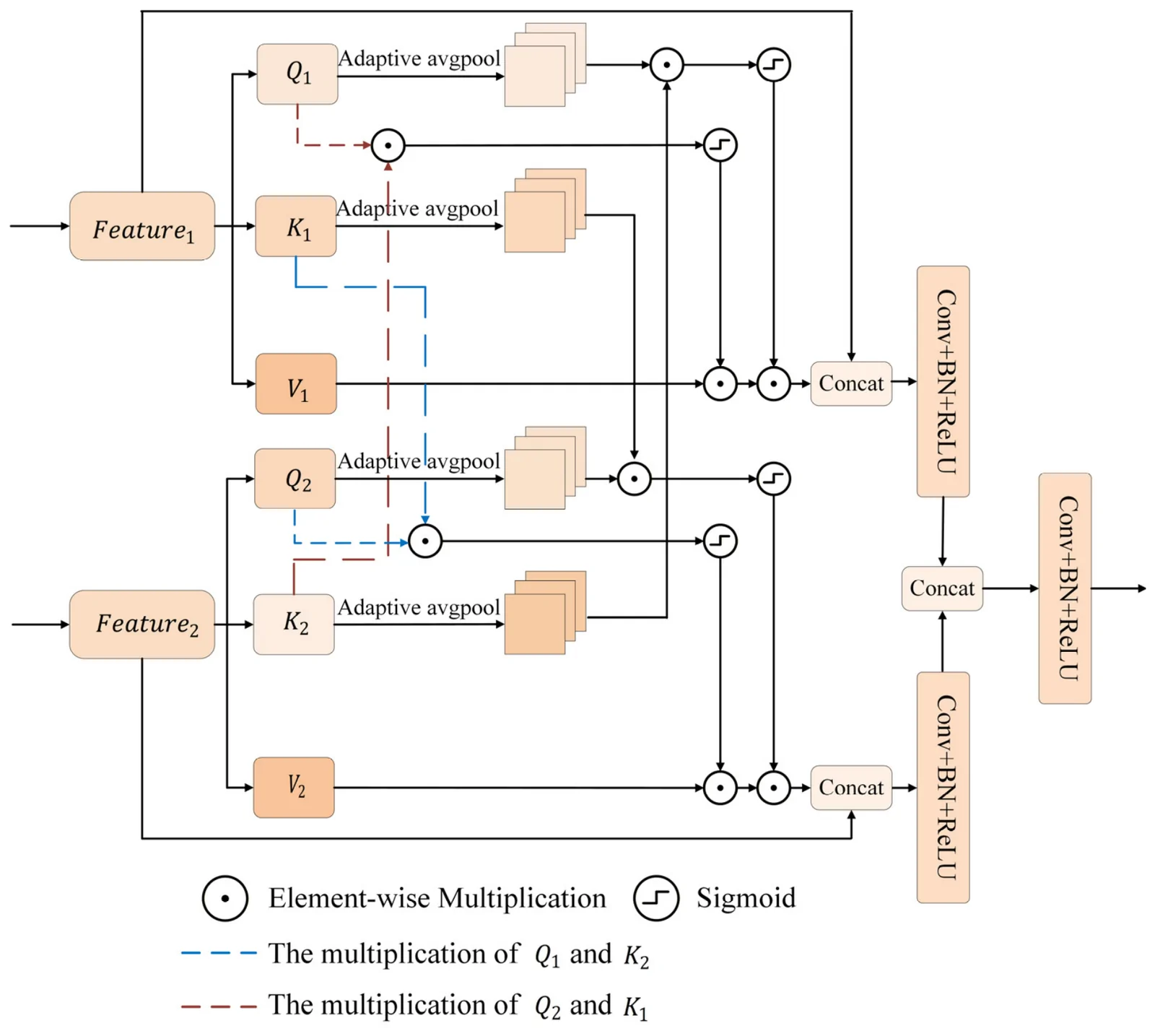
Architecture of the proposed Multi-Scale Cross-Modal Cooperative Fusion (MSCMCF) module. This module establishes bidirectional cross-modal attention to model inter-modality dependencies. It integrates channel and spatial attention to generate guidance weights, followed by a feature co-learning network and multi-scale depthwise separable convolutions to align semantic distributions and prevent modality dominance during fusion.

To address the challenges of semantic misalignment and insufficient exploitation of complementary information in multimodal medical image fusion, we design the Multi-Scale Cross-Modal Cooperative Fusion (MSCMCF) module, whose overall architecture is illustrated in Figure 2. Centered on a bidirectional cross-modal attention mechanism, the MSCMCF module integrates feature co-learning with adaptive alignment to enable deep, synergistic fusion of dual-modality features. Its core design comprises three key submodules: a cross-modal interaction attention module that dynamically models inter-modality dependencies, a feature co-learning network that jointly refines representations through mutual guidance, and an adaptive feature alignment layer that calibrates semantic discrepancies to ensure consistent and coherent fusion.

The design of the MSCMCF module is motivated by three key considerations. First, unlike unidirectional cross-attention schemes that allow information to flow from only one modality to the other, the proposed bidirectional attention pathways establish mutual guidance between modalities, ensuring that both contribute symmetrically to the fused representation and preventing either modality from dominating the fusion process. Second, the joint use of channel attention (CA) and spatial attention (SA) is driven by their complementary roles: CA identifies which semantic attributes are the most informative across modalities, while SA localizes where these attributes reside; their element-wise product ensures that both conditions must be satisfied for a feature to be enhanced, thereby producing more selective and reliable inter-modal correspondences than either mechanism alone could achieve. Third, the learnable enhancement weights are introduced to adaptively balance the original and cross-modal features rather than directly substituting the original representations, which prevents the degenerate case where cross-modal guidance overrides modality-specific information—such as lesion-specific metabolic signals in PET or SPECT—that is diagnostically essential and must be preserved in the fused output.

Within the cross-modal interaction attention module, the input bimodal feature maps are first projected via 1 × 1 convolutional layers into query (*Q*), key (*K*), and value (*V*) feature triplets, respectively, establishing a bidirectional attention flow. The computation is as defined in Equation (1):

(1) $$Q_{1},K_{1},V_{1}=Conv_{1\times 1}\left(F_{1}\right),Q_{2},K_{2},V_{2}=Conv_{1\times 1}\left(F_{2}\right)$$

where B, C, H, and W denote batch size, number of channels, feature height, and feature width, respectively.

Building upon this, to precisely capture cross-modal semantic correlations and generate inter-modal guidance weights, we construct two parallel attention pathways that jointly compute bidirectional attention weights by integrating channel attention (CA) and spatial attention (SA), as formulated in Equation (2):

(2) $$Attn_{1\to 2}=\sigma \left(SA\left(Q_{1},K_{2}\right)⊙CA\left(Q_{1},K_{2}\right)\right),Attn_{2\to 1}=\sigma \left(SA\left(Q_{2},K_{1}\right)⊙CA\left(Q_{2},K_{1}\right)\right)$$

where SA(⋅) denotes spatial attention, CA(⋅) denotes channel attention, σ(⋅) is the sigmoid activation function, and ⊙ represents element-wise multiplication.

Subsequently, the computed attention weights are applied to the corresponding value features to generate enhanced representations guided by the complementary modality, thereby enabling cross-modal information exchange and mutual enhancement. This feature refinement process is formalized in Equation (3):

(3) $$F_{1\to 2}=Attn_{1\to 2}\cdot V_{2},F_{2\to 1}=Attn_{2\to 1}\cdot V_{1}$$

Next, the feature co-learning network dynamically balances the contributions of the original and guidance features to prevent fusion results from being dominated by a single modality. This is achieved by concatenating the original and guided features and passing them through a lightweight convolutional subnetwork to generate learnable enhancement weights, as formulated in Equation (4):

(4) $$w_{1}=\sigma \left(ConvBlock\left(\left[F_{1},F_{1\to 2}\right]\right)\right),w_{2}=\sigma \left(ConvBlock\left(\left[F_{2},F_{2\to 1}\right]\right)\right)$$

where ConvBlock(⋅) denotes a basic convolutional block comprising a convolutional layer, batch normalization, and a ReLU activation, and $\left[\cdot ,\cdot \right]$ represents concatenation along the channel dimension.

To enable mutual enhancement of bimodal features based on the learned enhancement weights—preserving the dominant information of the original features while adaptively incorporating guidance from the complementary modality—we perform progressive feature refinement through cross-modal augmentation, as defined in Equation (5):

(5) $$F_{1}^{\prime }=F_{1}\cdot \left(1+w_{1}\right)+F_{1\to 2}\cdot w_{1},F_{2}^{\prime }=F_{2}\cdot \left(1+w_{2}\right)+F_{2\to 1}\cdot w_{2}$$

Finally, to align the spatial and semantic distributions of the bimodal features and mitigate cross-modal misalignment, we employ three parallel depthwise separable convolutional branches with kernel sizes of 3×3, 5×5, and 7×7, respectively. These kernels provide progressively enlarged spatial receptive fields: the 3×3 branch focuses on fine local structures and boundary details, while the 5×5 and 7×7 branches capture increasingly broader contextual information. Each branch consists of a channel-wise depthwise convolution followed by a 1×1 pointwise convolution for cross-channel interaction. All branches use a stride of 1, with padding values of 1, 2, and 3 for the 3×3, 5×5, and 7×7 convolutions, respectively, thereby preserving the spatial dimensions of the feature maps. The resulting multi-scale representations are aggregated to produce the aligned features, as formulated in Equation (6). Here, DSConv k(⋅) denotes a depthwise separable convolution with kernel size k×k, where k∈{3,5,7}.

(6) $$F_{1}^{\prime \prime }=\sum_{k=3,5,7}DWConv_{k}\left(F_{1}^{\prime }\right),F_{2}^{\prime \prime }=\sum_{k=3,5,7}DWConv_{k}\left(F_{2}^{\prime }\right)$$

where ${\mathrm{DWConv}}_{k}(\cdot )$ denotes a depthwise separable convolution with kernel size k.

Finally, to integrate the aligned bimodal features into a unified representation, we concatenate them and fuse them through a consolidation layer, while introducing a residual connection to enhance training stability. This yields a fused feature that synergistically combines the strengths of both modalities, as formalized in Equation (7):

(7) $$F_{out}=Conv_{fusion}\left(\left[F_{1}^{\prime \prime },F_{2}^{\prime \prime }\right]\right)+\alpha \cdot \frac{F_{1}+F_{2}}{2}$$

where ${\mathrm{Conv}}_{fusion}(\cdot )$ denotes a fusion convolutional layer, and α is a learnable residual weight parameter.

### 3.3. Multi-Scale Adaptive Fusion

**Figure 3:**
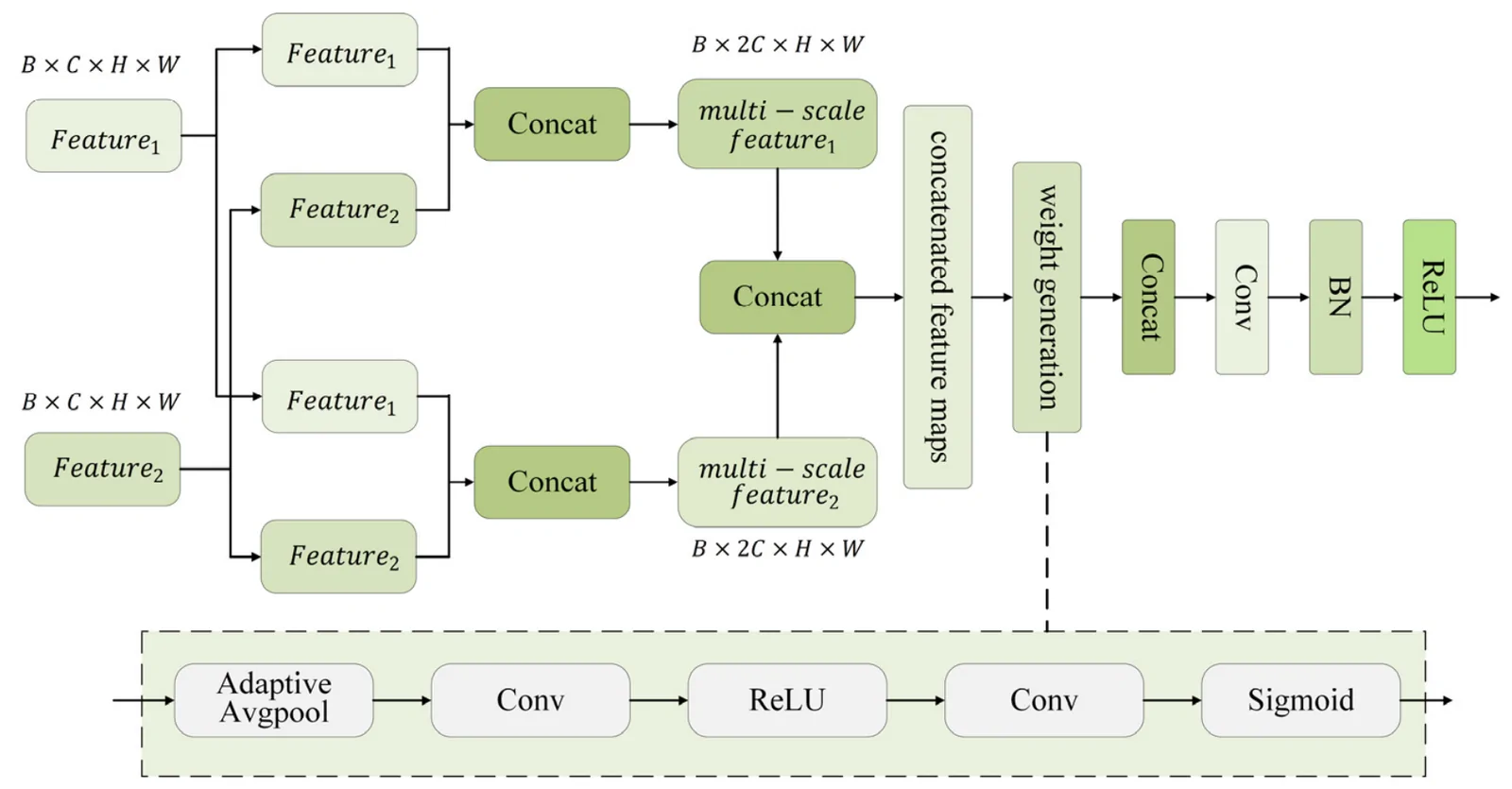
Architecture of the proposed Multi-Scale Adaptive Fusion (MSAF) module. The MSAF module utilizes a dual-scale feature extraction scheme (global 1 × 1 and local 2 × 2 adaptive pooling) to capture both semantic context and textural granularity. A lightweight weight prediction network dynamically generates normalized adaptive weights to balance the fusion of global and local features across modalities based on input content.

To fully exploit the complementary information embedded across multiple scales in multimodal medical images, we propose the Multi-Scale Adaptive Fusion (MSAF) module, whose overall architecture is illustrated in Figure 3. Built upon a dual-scale feature extraction scheme, the MSAF module integrates a lightweight adaptive weight prediction network to dynamically fuse features from different modalities and scales. This design enables context-aware balancing of local detail and global semantics—enhancing both fine-grained fidelity and structural coherence in the fused output—while maintaining computational efficiency through a compact, parameter-efficient architecture.

The MSAF module is designed with two principal motivations. First, the choice of a dual-scale scheme based on 1 × 1 and 2 × 2 adaptive average pooling is intended to achieve a compact yet complementary representation of global semantics and local spatial structure. Specifically, 1 × 1 adaptive average pooling aggregates the entire feature map into a global descriptor, thereby providing a holistic representation of modality-specific semantic information. However, using 1 × 1 pooling alone removes spatial variation and may overlook local anatomical boundaries and fine pathological structures. The additional 2 × 2 pooling branch preserves a coarse spatial partition of the feature map, enabling the network to retain regional intensity variations and textural information while maintaining low computational complexity. In comparison, finer pooling grids commonly used in spatial-pyramid designs, such as 3 × 3 and 4 × 4, produce 9 and 16 regional descriptors, respectively. Although these finer grids retain more spatial information, they also increase redundancy between neighboring pooled regions and enlarge the feature representation processed by the subsequent weight prediction network. They may also increase sensitivity to local noise and minor cross-modal misregistration. Therefore, 3 × 3 and 4 × 4 pooling were excluded, and the 1 × 1 and 2 × 2 configuration was adopted as a balanced trade-off among global context modeling, local detail preservation, and computational efficiency. Second, the adaptive weight prediction network is preferred over static fusion rules—such as simple averaging or fixed-ratio summation—because the optimal balance between global semantics and local details varies considerably across image regions and modality pairs; learnable, input-conditional weights allow the model to dynamically emphasize global structure in homogeneous tissue regions while prioritizing fine details near anatomical boundaries, yielding a context-sensitive fusion strategy that static rules cannot achieve.

First, to capture the complementary information between global semantics and local details, we perform dual-scale feature extraction on the input features, yielding a global-scale representation and a local-scale representation, as defined in Equation (8):

(8) $$F_{1}^{g},F_{1}^{l}={\mathrm{AdaptiveAvgPool}}_{1\times 1}\left(F_{1}\right),{\mathrm{AdaptiveAvgPool}}_{2\times 2}\left(F_{1}\right),\;F_{2}^{g},F_{2}^{l}={\mathrm{AdaptiveAvgPool}}_{1\times 1}\left(F_{2}\right),{\mathrm{AdaptiveAvgPool}}_{2\times 2}\left(F_{2}\right)$$

where B, C, H, and W denote batch size, number of channels, feature height, and feature width, respectively; the superscript g indicates the global-scale feature, and l denotes the local-scale feature.

To integrate multi-scale, multimodal information and provide a comprehensive feature representation for subsequent weight prediction, we concatenate the multi-scale features from both modalities along the channel dimension, as formulated in Equation (9):

(9) $$F_{ms_{1}}=\mathrm{Concat}\left(F_{1}^{g},F_{1}^{l}\right),F_{ms_{2}}=\mathrm{Concat}\left(F_{2}^{g},F_{2}^{l}\right),F_{cat}=\mathrm{Concat}\left(F_{ms_{1}},F_{ms_{2}}\right)$$

where Concat(⋅) denotes concatenation along the channel dimension, $F_{ms_{1}}$ and $F_{ms_{2}}$ represent the multi-scale features from the two modalities, respectively, and $F_{\mathrm{cat}}\in R^{B\times 4C\times H\times W}$ is the resulting concatenated feature tensor.

To dynamically learn importance weights for features across scales and modalities, we process the concatenated feature tensor through a lightweight weight prediction network, producing normalized adaptive weights as defined in Equation (10):

(10) $$w=\sigma \left({\mathrm{Conv}}_{1\times 1}\left(\mathrm{ReLU}\left({\mathrm{Conv}}_{1\times 1}\left(\mathrm{GAP}\left(F_{cat}\right)\right)\right)\right)\right)\in R^{B\times 2C\times 1\times 1}$$

where GAP(⋅) denotes global average pooling, and σ(⋅) is the Sigmoid activation function, whose output lies in the interval $\left[0,1\right]$, thereby satisfying the requirement that modality-specific contribution weights be bounded and interpretable.

Intuitively, this weight prediction mechanism functions as a context-aware routing system. The global average pooling operation compresses the spatial dimensions to extract a global semantic descriptor, while the subsequent Sigmoid activation maps this descriptor into a normalized importance score. Conceptually, these adaptive weights dictate the network’s focus: in homogeneous tissue regions where global semantic consistency is paramount, the network assigns higher weights to the global-scale features (1 × 1 pooling); conversely, near anatomical boundaries or fine pathological structures, the weights shift to prioritize local-scale features (2 × 2 pooling) to preserve textural granularity. This dynamic balancing directly influences the overall fusion performance by preventing the over-smoothing of critical details—a common failure in static fusion rules—thereby ensuring that the fused output maintains both structural coherence and high-fidelity local details. For a clear visual representation of this process, readers are referred to Figure 3, which illustrates the complete data flow from dual-scale extraction to the final adaptive weighted fusion.

The weight tensor is then split into global-scale weights $w_{g}$ and local-scale weights $w_{l}$, which are applied to the corresponding scale-specific bimodal features to perform weighted fusion, as formulated in Equation (11):

(11) $$\;F_{g}=w_{g}\cdot F_{1}^{g}+\left(1-w_{g}\right)\cdot F_{2}^{g},\;F_{l}=w_{l}\cdot F_{1}^{l}+\left(1-w_{l}\right)\cdot F_{2}^{l}$$

To integrate the multi-scale fused features and refine their distribution, we concatenate the global- and local-scale fused features and process them through a convolutional layer followed by batch normalization, as formulated in Equation (12):

(12) $$F_{fused}=\mathrm{ConvBlock}\left(\mathrm{Concat}\left(F_{g},F_{l}\right)\right)\in R^{B\times C\times H\times W}$$

where ConvBlock(⋅) denotes a basic convolutional block comprising a 3×3  convolution, batch normalization, and a ReLU activation.

To preserve fine-grained details from the original features and enhance training stability, we introduce a learnable residual weight β and incorporate the mean of the original bimodal features as a residual term into the fused output, as formulated in Equation (13):

(13) $$F_{out}=F_{fused}+\beta \cdot \frac{F_{1}+F_{2}}{2}$$

### 3.4. Vision RWKV Block

The Receptance Weighted Key Value (RWKV) architecture was introduced by Peng et al. [48] as a sequence model that combines the parallelizable training of Transformers with the constant memory, linear time inference of recurrent neural networks. RWKV reformulates the $softmax\left(QK⊤\right)V$ self-attention as a channel-wise linear attention, in which a learnable time decay coefficient w replaces the pairwise interaction term. Concretely, the WKV operator is derived from Attention-Free Transformers (AFT) [4] by replacing AFT’s learned scalar pairwise bias $w_{\left\{t,i\right\}}$ with a closed-form exponential decay $w_{\left\{t,i\right\}}=-\left(t-i\right)w$, which can be summed as a linear recurrence and thus admits both a parallel formulation during training and an RNN-style recurrence during inference. Each block contains three components: a token shift (a channel-wise linear interpolation between $x_{t}\;and\;x_{\left(t-1\right)})$, the WKV operator, and an output gate $\sigma \left(r_{t}\right)$. Because the pairwise T × T attention matrix is eliminated, RWKV achieves O(Td) time and O(d) state complexity per step ([48]), in contrast with the $O\left(T^{2}d\right)$ time and $O\left(T^{2}+Td\right)$ memory of standard self-attention. This exact, non-approximate linear attention has recently shown competitive performance on volumetric medical image segmentation [49], motivating our extension to 2D spatial modeling in the VRWKV block.

The choice of RWKV as the global spatial modeling backbone, rather than alternative efficient attention mechanisms, is based on three considerations. First, unlike Linformer and Performer, which approximate self-attention through low-rank or kernel-based approximations that can degrade representation fidelity at high resolutions, RWKV achieves exact linear complexity through a recurrent formulation with no approximation error, making it particularly suitable for medical images where precise spatial relationships carry clinical significance. Second, RWKV’s bidirectional attention variant (Bi-WKV) naturally models both forward and backward spatial dependencies, which is essential for capturing the symmetric anatomical structures frequently encountered in medical images—such as bilateral organs and contralateral brain hemispheres—that unidirectional recurrent models would process asymmetrically. Third, the adaptive local shifting operation is introduced to explicitly expand the local receptive field before global attention computation; without this shift, the linear attention kernel would attend with equal weight across all spatial positions, diluting the contribution of nearby pixels that carry strong local correlations (e.g., edges, textures, and fine anatomical boundaries) and are particularly informative for preserving structural detail in the fused output.

**Figure 4:**
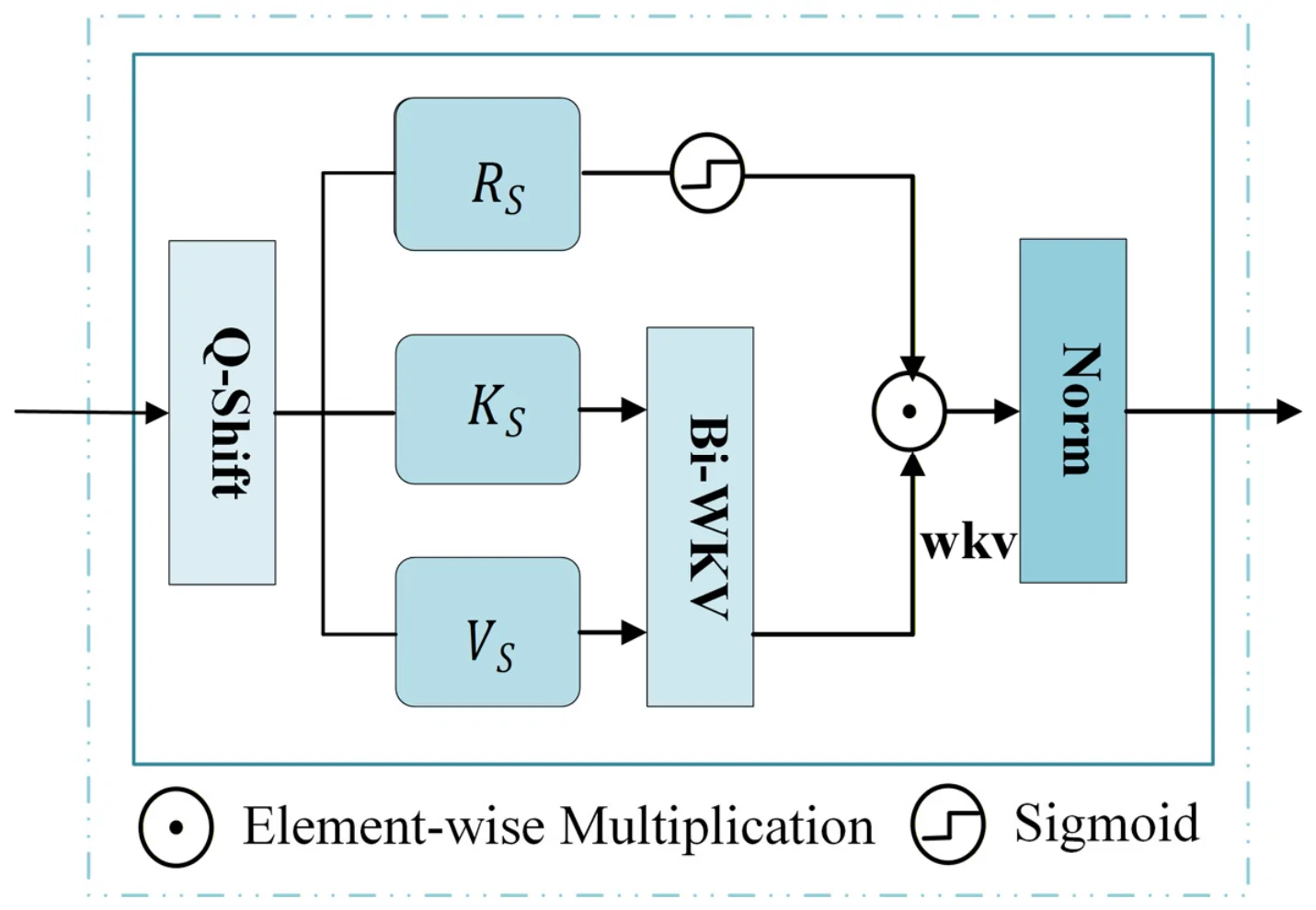
Architecture of the proposed Vision RWKV (VRWKV) Block. The block reshapes 2D features into sequences and applies an adaptive local shifting operation to expand the receptive field. It then projects features into Receptance, Key, and Value triplets, utilizing a bidirectional linear attention (Bi-WKV) mechanism with spatial decay to efficiently model long-range spatial dependencies with exact O(T) linear complexity, followed by a gated residual connection to preserve fine details.

As shown in Figure 4, first, to expand the local receptive field and enhance the representation of edges and fine textures, we apply a deterministic, channel-partitioned pixel shifting operation to the input feature map before sequence flattening. The channel dimension is partitioned into five groups, denoted as *Xu*, *Xd*, *Xl*, *Xr*, and *X*0. The first four groups are assigned to the upward, downward, leftward, and rightward shifting directions, respectively, with each group containing m channels. The remaining channels constitute an identity group X0, which remains unshifted. The shifted feature map is formulated in Equation (14).

(14) $$X_{\mathrm{shift}}=\mathrm{Reshape}\left(\mathrm{Concat}\left(S_{u}^{s}\left(X^{u}\right),S_{d}^{s}\left(X^{d}\right),S_{l}^{s}\left(X^{l}\right),S_{r}^{s}\left(X^{r}\right),X^{0}\right)\right)$$

Specifically, m is calculated as the integer part of the shift ratio multiplied by the total number of channels *C*. The operators *Su*, *Sd*, *Sl*, and *Sr* denote the spatial shifting operations along the upward, downward, leftward, and rightward directions by s pixels, respectively. The newly exposed boundary positions are padded with zeros, and no circular wrapping is applied. Consequently, the shifting direction for each channel is strictly determined by its channel index and remains invariant during both training and inference. Following the spatial shifting and concatenation, the feature map is reshaped into a sequence of length T, where T equals H multiplied by W, resulting in the final output dimension of B by T by C for subsequent projection and bidirectional linear attention.

To establish a normalized foundational representation for subsequent linear attention computation, we project the shifted features into query (Receptance, R), key (K), and value (V) triplets via channel-wise linear projection layers, as formulated in Equation (15):

(15) $$R=\mathrm{Linear}\left(X_{shift}\right),K=\mathrm{Linear}\left(X_{shift}\right),V=\mathrm{Linear}\left(X_{shift}\right)$$

where Linear(⋅) denotes a linear projection that preserves the feature dimensionality to match the input, thereby ensuring dimensional compatibility for subsequent attention computation.

The core of the VRWKV block is bidirectional linear attention (Bi-WKV). To enable efficient aggregation of global contextual information while preserving linear computational complexity, we introduce spatial decay coefficients and initial state weights to modulate the contribution of features at different spatial positions, as formulated in Equation (16):

(16) $$Y_{t}=u\cdot V_{t}+\sum_{i=1}^{t-1}e^{w\cdot \left(i-t\right)}\cdot K_{i}\cdot V_{i}$$

where t denotes the sequence position index, $w\in R^{C}\;$is a learnable spatial decay parameter that modulates the contribution of distant features to suppress irrelevant information, and $u\in R^{C\;}$ is an initial weight parameter that balances the influence of the current position against its historical context; the term $e^{w\cdot (i-t)}$ serves as an exponential decay factor that ensures the overall attention computation remains O(T)—i.e., linear in sequence length—making it scalable to high-resolution medical images.

To enhance the nonlinearity of feature representations and enable adaptive selection of the aggregated global contextual features, we apply element-wise multiplication between the attention output and the query (Receptance, R) via a Sigmoid gating mechanism, as formulated in Equation (17):

(17) $$Y_{\mathrm{gate}}=\sigma \left(R\right)⊙Y$$

where σ(⋅) denotes the Sigmoid activation function and ⊙ represents element-wise multiplication; this gating mechanism adaptively selects informative aggregated features based on the intrinsic content of the input while suppressing redundant or less relevant components.

Finally, to restore the gated features to the original input dimensionality and improve training stability, we apply a linear projection followed by a residual connection, as formulated in Equation (18):

(18) $$X_{out}=\mathrm{Linear}\left(Y_{gate}\right)+X_{seq}$$

The flattened sequence features are then reshaped back into a 2D spatial format $X_{\mathrm{out}}\in R^{B\times C\times H\times W}$, and a learnable weighting parameter is introduced to balance the contribution of the residual branch—thereby preserving fine-grained details from the original features while effectively integrating the semantic information from the globally aggregated context.

## 4. Experiments

### 4.1. Datasets, Implementation Details, and Evaluation Metrics

#### 4.1.1. Datasets

In this study, we adopt the Harvard Medical Dataset as our primary data source, which provides image pairs for three canonical multimodal medical image fusion tasks: CT–MRI, PET–MRI, and SPECT–MRI. This dataset comprehensively supports the training and evaluation of multimodal fusion models by offering complementary anatomical and functional imaging modalities. Specifically, it contains 166 CT–MRI pairs, 329 PET–MRI pairs, and 539 SPECT–MRI pairs. To minimize interference from input size heterogeneity, all images are uniformly cropped to a standard resolution of 256 × 256 pixels, ensuring consistent input dimensions and streamlining the preprocessing pipeline. For reliable and reproducible evaluation, we employ a 5-fold cross-validation strategy on each modality-specific subset, thereby enabling a robust assessment of model performance and generalization capability.

The Brain Tumor Segmentation (BraTS) [50] dataset is a widely recognized benchmark providing multi-parametric magnetic resonance imaging (MRI) scans for brain tumor analysis. In this study, we utilize the Fluid-Attenuated Inversion Recovery (FLAIR) and T1 contrast-enhanced (T1ce) modalities from this dataset to formulate our image fusion task. Specifically, a total of 1251 2D slices are extracted from these sequences. To ensure rigorous evaluation, a 5-fold cross-validation protocol is similarly adopted to comprehensively assess the proposed fusion framework on the BraTS dataset.

#### 4.1.2. Implementation Details

All experiments in this study are implemented using PyTorch 2.1.2 and executed on an NVIDIA GeForce RTX 4090 GPU with 24 GB memory. To account for the distinct imaging characteristics across modalities, we adopt modality-specific preprocessing strategies. For the CT–MRI fusion task, both source images are grayscale and thus directly fed into the model without any color–space conversion. In contrast, for PET–MRI and SPECT–MRI tasks, PET and SPECT images are provided in RGB format. Following the color–space transformation protocol established in [23,51], we first convert these images to the YCbCr color–space. Only the Y channel, which encodes luminance and structural information critical for medical interpretation, is used as an input to the fusion model. Before being fed into the network, all grayscale images and extracted Y channels were converted to single-precision floating-point tensors and normalized to an intensity range from zero to one. The same fixed-range normalization procedure was consistently applied to all patients and imaging modalities. No Z-score standardization or patient-specific, image-specific, or modality-specific statistical normalization was performed. This preprocessing ensured a consistent numerical range for network training while preserving the relative intensity distribution of the source images. The chrominance channels (($C_{b}$ and $C_{r}$), which carry color information lacking diagnostic relevance in nuclear medicine, are preserved separately but excluded from training; they are stored independently while maintaining strict spatial alignment with their corresponding Y channels. After the fusion network generates a fused Y channel by integrating the PET/SPECT Y channel with the MRI image, we reconstruct the final RGB output by recombining the fused Y channel with the original, unmodified $C_{b}$ and $C_{r}$ channels from the respective PET or SPECT image, followed by an inverse YCbCr-to-RGB transformation. This strategy effectively decouples structural and chromatic information: it ensures that cross-modal fusion operates solely on diagnostically meaningful features while preserving the original color appearance for visual interpretability. Consequently, the resulting fused images retain both high-fidelity anatomical–functional structure and natural color representation, enhancing clinical usability without compromising fusion performance.

Training Strategy: All models are trained using the AdamW optimizer with an initial learning rate of $1\times 10^{-3}$. The training is conducted for 100 epochs with a batch size of 2, without learning rate scheduling or warmup, ensuring consistent optimization conditions across all ablation and comparison experiments.

Loss Function: Following the well-established composite loss formulation in [23], our training objective is designed to guarantee visual fidelity and appropriate intensity information from multiple aspects. Specifically, the total loss LL is defined as a weighted sum of three components: the structural similarity index measure (SSIM) loss (Lssim), the gradient/texture loss (Lgrad), and the intensity loss (Lint), formulated as: L = δ1Lssim + δ2Lgrad + δ3Lint where *δ*1, *δ*2, and *δ*3 are the trade-off weights. In our implementation, we set *δ*1 = 1, *δ*2 = 10, and *δ*3 = 10. The rationale for this configuration is based on the inherent scale differences among the loss components. The SSIM loss typically yields values in the range of [0, 1], whereas the gradient and intensity losses, computed via pixel-wise absolute differences and Sobel operators, generally produce much smaller numerical magnitudes. Assigning larger weights to the gradient and intensity losses effectively balances their gradient contributions during backpropagation, preventing the optimization from being dominated by the SSIM term and ensuring the simultaneous preservation of structural fidelity, fine-grained textures, and critical intensity contrasts. This setting aligns with the empirical conventions in the medical image fusion community and was validated in our preliminary experiments to yield optimal performance.

#### 4.1.3. Evaluation Metrics

To quantitatively evaluate the performance of the proposed method, we employ six widely adopted metrics in medical image fusion: mutual information (MI), mean squared error (MSE), correlation coefficient (CC), peak signal-to-noise ratio (PSNR), structural similarity index (SSIM), and structural content difference (SCD):

Mutual information (MI) quantifies the amount of shared information between the fused image F and each source modality, computed based on their joint probability distribution, as defined in Equation (19):

(19) $$\;MI\left(F,A\right)=\sum_{f}\sum_{a}p\left(f,a\right){log}_{2}\frac{p\left(f,a\right)}{p\left(f\right)p\left(a\right)},MI_{\mathrm{total}}=\mathrm{MI}\left(F,A\right)+\mathrm{MI}\left(F,B\right)$$

Mean squared error (MSE) measures the average squared difference between corresponding pixels of the fused image and the structural modality source image, as formulated in Equation (20):

(20) $$\mathrm{MSE}=\frac{1}{MN}\sum_{i=1}^{M}\sum_{j=1}^{N}{\left[F\left(i,j\right)-A\left(i,j\right)\right]}^{2}$$

The correlation coefficient (CC) quantifies the linear correlation between the fused image and the source image in terms of structural patterns and intensity variations, as defined in Equation (21):

(21) $$\mathrm{CC}\left(F,A\right)=\frac{\sum_{i,j}\left[F\left(i,j\right)-\mu_{F}\right]\left[A\left(i,j\right)-\mu_{A}\right]}{\sqrt{\sum_{i,j}{\left[F\left(i,j\right)-\mu_{F}\right]}^{2}\sum_{i,j}{\left[A\left(i,j\right)-\mu_{A}\right]}^{2}}}$$

Peak signal-to-noise ratio (PSNR) quantifies the distortion between the fused image and the source image, computed from the mean squared error as defined in Equation (22):

(22) $$\mathrm{PSNR}=10\log_{10}\left(\frac{L^{2}}{\mathrm{MSE}}\right)$$

The structural similarity index (SSIM) evaluates the perceptual consistency between the fused image and the source image by jointly considering luminance, contrast, and structural information, as formulated in Equation (23):

(23) $$\mathrm{SSIM}\left(x,y\right)=\frac{\left(2\mu_{x}\mu_{y}+c_{1}\right)\left(2\sigma_{xy}+c_{2}\right)}{\left(\mu_{x}^{2}+\mu_{y}^{2}+c_{1}\right)\left(\sigma_{x}^{2}+\sigma_{y}^{2}+c_{2}\right)}$$

Structural content difference (SCD) quantifies the discrepancy in high-frequency structural content (e.g., edges and fine textures) between the fused image and the source images, as defined in Equation (24). Unlike SSIM, which evaluates macro-structural similarity and luminance, SCD explicitly isolates high-frequency details. This makes it highly relevant for clinical tasks requiring precise boundary delineation, such as tumor contouring, where the preservation of fine anatomical edges is critical.

(24) $$\mathrm{SCD}=\left|\bar{SI_{A}}-\bar{SI_{F}}\right|+\left|\bar{SI_{B}}-\bar{SI_{F}}\right|,SI=\sqrt{{\left(\nabla_{x}I\right)}^{2}+{\left(\nabla_{y}I\right)}^{2}}$$

### 4.2. Comparison with State-of-the-Art Methods

To rigorously evaluate the effectiveness of the proposed method, we conduct a comparative study against seven state-of-the-art multimodal medical image fusion approaches: MACTFusion [23], GeSeNet [15], FATFusion [26], MMIFINet [52], IFCNN [39], U2Fusion [53], and SwinFusion [25]. All comparative experiments are evaluated using a strict 5-fold cross-validation protocol to ensure the robustness and reliability of the reported quantitative results.

#### 4.2.1. CT–MRI Dataset

Quantitative results on the CT–MRI dataset are presented in Table 1. The compared methods exhibit different strengths across the evaluation metrics. GeSeNet achieves competitive performance in MI, MSE, and PSNR, while U2Fusion obtains a relatively high CC and low MSE. MMIFINet and SwinFusion perform well in SSIM, indicating effective structural information preservation. However, these methods show less balanced performance across the remaining metrics.

Our method achieves the best performance on five of the six evaluation metrics. Specifically, it attains the highest MI of 0.9146, indicating effective preservation of complementary information from the source modalities. It also obtains the lowest MSE of 0.0423 and the highest PSNR of 15.1485 dB, outperforming the second-best GeSeNet results of 0.0436 and 14.9578 dB, respectively. These results demonstrate reduced pixel-level reconstruction error and improved image fidelity. Moreover, the highest CC of 0.8198 and SCD of 0.7023 further confirm the method’s ability to preserve cross-modal feature correlation and structural information.

**Figure 5:**
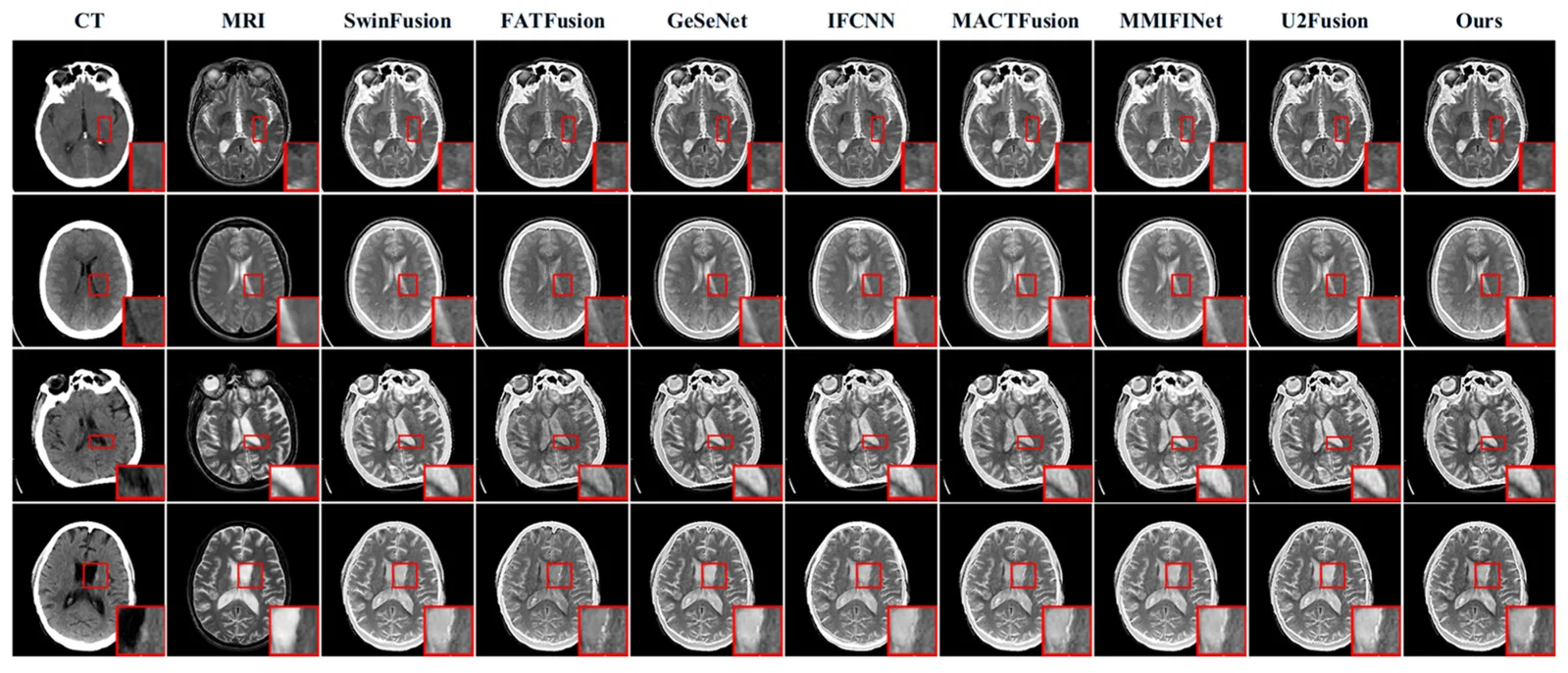
Qualitative comparison of fusion results on the CT–MRI dataset. The columns display the source CT, source MRI, and the fused results from seven state-of-the-art methods alongside our proposed MSFusion. Zoomed-in regions (highlighted by colored boxes) demonstrate that MSFusion preserves sharper anatomical boundaries and finer textural details (e.g., bone structures and soft tissue interfaces) with fewer artifacts compared to competing methods, corroborating its superior quantitative performance in MSE and PSNR.

Although MSFusion achieves a lower SSIM than the competing methods, this result should be interpreted together with the other quantitative and qualitative findings. SSIM evaluates similarity in luminance, contrast, and relatively large-scale structural patterns with respect to a source image. In multimodal fusion, however, the fused image is expected to integrate complementary information from both CT and MRI rather than reproduce the appearance of either source modality. The incorporation of modality-specific information, local contrast variations, and sharpened anatomical boundaries may therefore reduce source-oriented SSIM even when diagnostically relevant details are better preserved. This interpretation is supported by the highest SCD obtained by MSFusion, which indicates superior preservation of high-frequency structural content, including edges and fine textures. The visual results in Figure 5 similarly show sharper bone boundaries and soft tissue interfaces with fewer blurring artifacts. Therefore, the relatively lower SSIM may reflect a trade-off between macro-structural similarity and the preservation of complementary high-frequency details. The bidirectional linear attention in the VRWKV block may also redistribute contextual information over long spatial ranges, resulting in local intensity and contrast differences from the individual source images. However, the lower SSIM cannot be attributed solely to the VRWKV block without a dedicated component-level analysis. Overall, the superior MI, MSE, CC, PSNR, and SCD results indicate that MSFusion achieves a favorable balance between complementary information transfer, reconstruction fidelity, global dependency modeling, and fine detail preservation.

#### 4.2.2. PET–MRI Dataset

Quantitative results on the PET–MRI dataset are presented in Table 2. The compared methods exhibit different strengths across the evaluation metrics. GeSeNet and U2Fusion achieve competitive MI values, while MMIFINet and SwinFusion perform well in SSIM. However, these methods show less favorable performance in reconstruction accuracy or overall image fidelity. In particular, IFCNN obtains the lowest PSNR among the compared methods, indicating relatively limited detail preservation.

Our method achieves the best performance on five of the six evaluation metrics. Specifically, it attains the highest MI of 1.2612, slightly surpassing GeSeNet at 1.2578, demonstrating effective preservation of complementary information from the source modalities. It also obtains the lowest MSE of 0.0524 and the highest PSNR of 16.9628 dB, indicating reduced pixel-level reconstruction error and improved image fidelity. Moreover, the highest CC of 0.7851 and SCD of 0.6537 further demonstrate the method’s ability to preserve cross-modal feature correlation and structural information.

**Figure 6:**
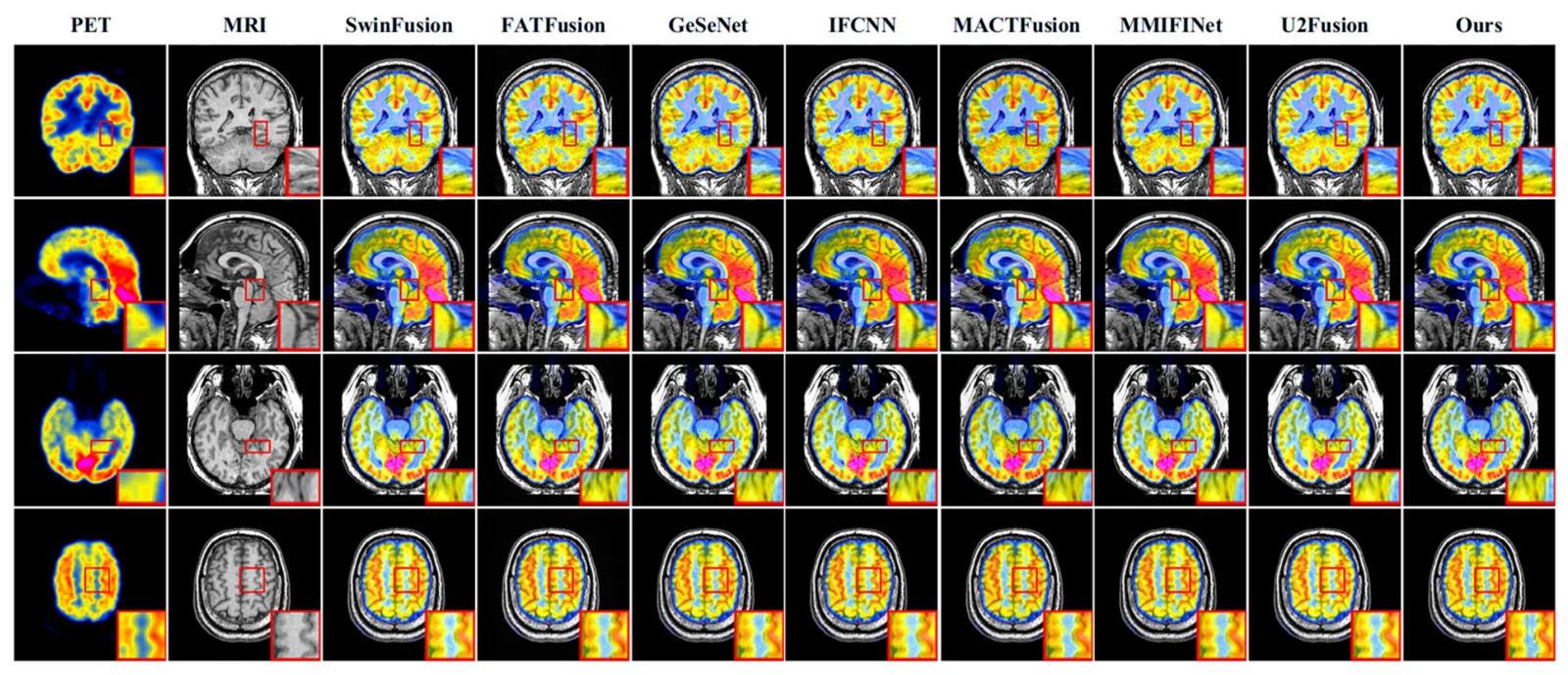
Qualitative comparison of fusion results on the PET–MRI dataset. Visual comparisons highlight the integration of functional metabolic information (from PET) with high-resolution anatomical structures (from MRI). The proposed MSFusion effectively retains the intensity and spatial localization of metabolic hotspots while maintaining clear anatomical contours, outperforming baselines that exhibit either color distortion, structural blurring, or loss of functional details. The red box indicates local zoomed-in details.

Although the SSIM of our method is lower than that of several competing approaches, its superior performance in MI, MSE, CC, PSNR, and SCD demonstrates a favorable overall balance among information transfer, reconstruction accuracy, detail preservation, and structural consistency. These results validate the effectiveness of the proposed method for PET–MRI multimodal image fusion. The qualitative comparisons shown in Figure 6 further support these findings.

#### 4.2.3. SPECT–MRI Dataset

Quantitative results on the SPECT–MRI dataset are presented in Table 3. The compared methods exhibit different strengths across the evaluation metrics. SwinFusion achieves the highest SSIM among the baseline methods, while GeSeNet, MMIFINet, and IFCNN show competitive performance in selected structural and fidelity-related metrics. U2Fusion obtains a relatively high MI value but performs poorly in MSE, CC, PSNR, SSIM, and SCD, indicating limited reconstruction accuracy and structural consistency.

Our method achieves the best performance on five of the six evaluation metrics. Specifically, it obtains the highest MI of 1.3021, slightly surpassing U2Fusion at 1.2978, indicating effective preservation of complementary information from the source modalities. It also achieves the lowest MSE of 0.0135 and the highest PSNR of 21.9846 dB, demonstrating reduced pixel-level reconstruction error and improved image fidelity. Moreover, our method attains the highest CC of 0.8920 and SCD of 0.4041, confirming its ability to preserve feature correlation and structural information.

**Figure 7:**
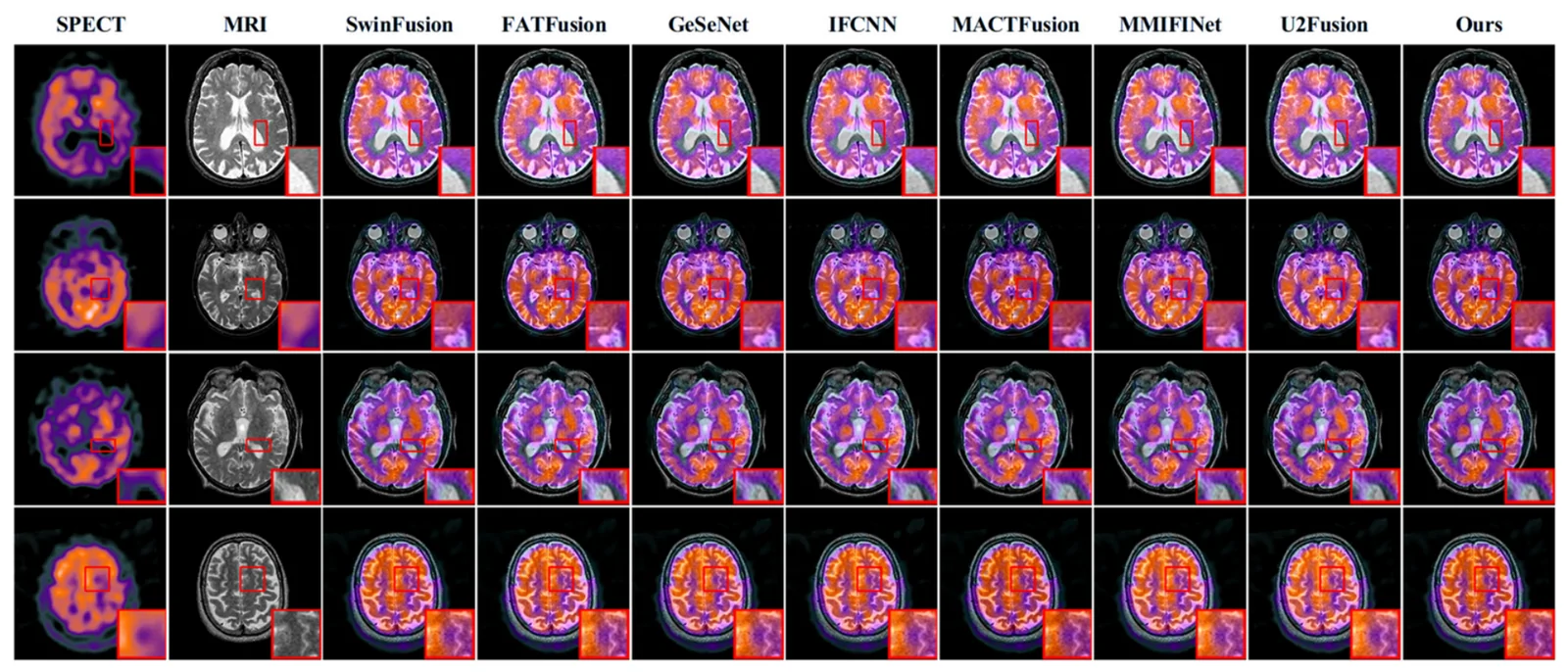
Qualitative comparison of fusion results on the SPECT–MRI dataset. Given the low-contrast and noise-prone nature of SPECT images, this comparison focuses on the preservation of functional activity regions against the anatomical background. MSFusion demonstrates superior capability in suppressing background noise while clearly delineating the functional uptake regions, avoiding the over-smoothing or severe artifact introduction seen in other methods.

Although the SSIM of our method is lower than that of several competing approaches, its superior performance in MI, MSE, CC, PSNR, and SCD demonstrates a favorable overall balance among information transfer, reconstruction accuracy, image fidelity, and structural preservation. These results validate the effectiveness of the proposed MSFusion framework for SPECT–MRI multimodal image fusion. The qualitative comparisons shown in Figure 7 further support these findings.

#### 4.2.4. Flair-T1ce Dataset

As shown in Table 4, the experiments are conducted on the BraTS (Brain Tumor Segmentation) dataset, which is widely used for multimodal brain MRI analysis. In this study, we utilize the FLAIR and T1ce modalities to evaluate the performance of different fusion methods. The FLAIR modality highlights edema and lesion regions, while the T1ce modality emphasizes tumor-enhanced areas after contrast injection. These two modalities provide complementary information, making them suitable for image fusion tasks.

A total of 251 paired Flair-T1ce images are selected for evaluation. All images are preprocessed to ensure spatial alignment and intensity normalization. The fusion performance is quantitatively assessed using multiple metrics, including mutual information (MI), mean squared error (MSE), correlation coefficient (CC), peak signal-to-noise ratio (PSNR), structural similarity index (SSIM), and structural content difference (SCD).

#### 4.2.5. Scussion of Comparative Results

The quantitative results across the three benchmark datasets reveal several consistent patterns that contextualize the contribution of MSFusion relative to existing approaches. First, no competing method achieves top-tier performance uniformly across all three modality pairs: CNN-centric methods such as GeSeNet attain strong mutual information scores on PET–MRI (MI = 1.2596) owing to their effective local feature extraction, yet exhibit elevated MSE on SPECT–MRI (0.0144), reflecting their limited capacity to model global anatomical context under low-contrast conditions. Transformer-based methods such as SwinFusion achieve a balanced profile across metrics but are constrained by the limited medical imaging data regime (80–430 training pairs per modality), which is insufficient to fully exploit the data-hungry nature of self-attention mechanisms. Unsupervised approaches such as U2Fusion, while avoiding the need for paired training data, suffer catastrophic degradation on the challenging SPECT–MRI task (MSE = 0.0446, SSIM = 0.5498), underscoring the importance of supervised cross-modal alignment when source image quality is low. In contrast, MSFusion achieves the best or near-best performance across the clinically critical metrics of MSE, PSNR, and CC on all three datasets, and is the only method that maintains robust performance on SPECT–MRI—the most challenging fusion scenario—without sacrificing CT–MRI or PET–MRI quality. We attribute this cross-modal robustness to the synergistic combination of MSCMCF, which enforces explicit cross-modal semantic alignment; MSAF, which dynamically balances multi-scale contributions through input-conditional weighting; and VRWKV, which captures long-range spatial dependencies with linear complexity, together enabling the model to adapt its fusion strategy to the specific characteristics of each modality pair rather than applying a uniform fusion rule. These findings collectively indicate that MSFusion’s hierarchical, adaptive fusion design offers a meaningful advantage over both purely local (CNN) and purely global (Transformer) paradigms, particularly in clinically realistic scenarios where source image quality and contrast vary considerably across modalities.

**Figure 8:**
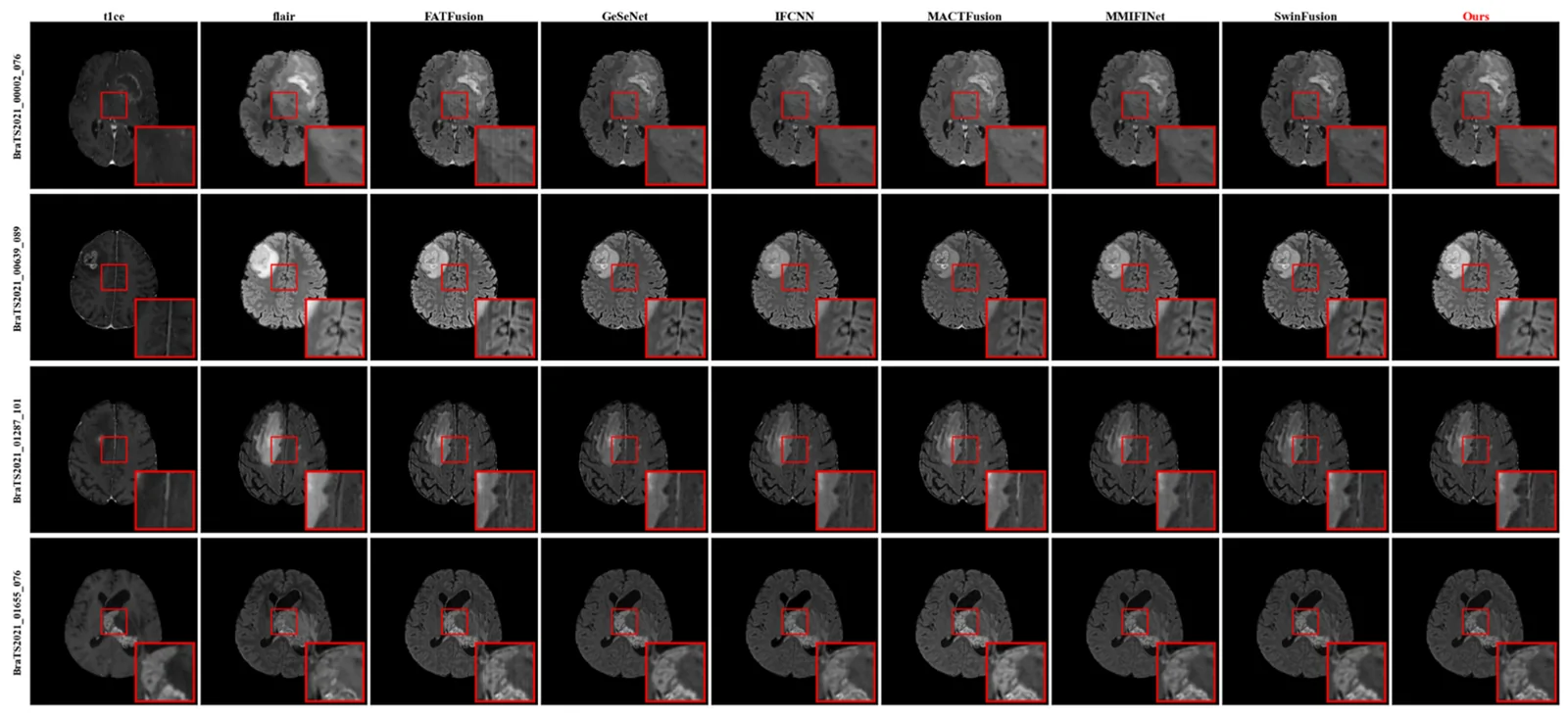
Qualitative comparison of fusion results on the Flair-T1ce dataset. The first two columns display the source T1ce and FLAIR images, respectively. The subsequent columns show the fused results from six state-of-the-art methods (FATFusion, GeSeNet, ECNN, MACTFusion, MMIFINet, and SwinFusion) alongside our proposed MSFusion (ours). The red box indicates local zoomed-in details.

A more detailed examination of the representative qualitative results provides additional insight into the potential clinical relevance of the fused images. In the CT–MRI examples shown in Figure 5, MSFusion preserves the sharp cortical boundaries of high-density bone structures while maintaining the adjacent soft tissue interfaces derived from MRI. Several competing methods exhibit either blurred bone margins or reduced soft tissue contrast in the enlarged regions, whereas MSFusion retains clearer transitions between these structures with fewer visible fusion artifacts. In the PET–MRI examples shown in Figure 6, the proposed method preserves both the intensity and spatial localization of metabolically active regions from PET while maintaining the surrounding anatomical contours provided by MRI. This is important because excessive smoothing may enlarge or weaken a metabolic hotspot, whereas color or intensity distortion may alter its apparent functional significance. In the SPECT–MRI examples shown in Figure 7, MSFusion more clearly delineates functional uptake regions against the low-contrast anatomical background and suppresses diffuse background noise without eliminating the uptake signal. In the FLAIR–T1ce examples shown in Figure 8, the fused images maintain the broader hyperintense lesion-related regions visible in FLAIR together with the more localized contrast-enhancing structures visible in T1ce, while preserving the boundaries between abnormal and surrounding tissues. These observations suggest that the distributed multi-scale fusion strategy is effective in preserving clinically relevant structures with different spatial scales and contrast characteristics, including bone boundaries, soft tissue interfaces, functional hotspots, uptake regions, lesion-related hyperintensity, and contrast-enhancing tumor regions.

These structure-specific qualitative observations provide complementary evidence for the potential clinical value of MSFusion by demonstrating improved preservation of diagnostically relevant anatomical and functional features across different modality combinations. In particular, the clearer delineation of lesion boundaries, metabolic hotspots, functional uptake regions, and surrounding tissue interfaces suggests that the fused images may support more reliable visual interpretation and lesion localization. Although a formal blinded reader study involving board-certified radiologists was beyond the scope of the present work, the consistent improvements observed across quantitative metrics and representative pathological structures provide a strong basis for further clinical validation. Future work will therefore extend the current evaluation through randomized, anonymized, multi-reader studies to assess diagnostic confidence, lesion conspicuity, artifact severity, and inter-reader agreement.

Furthermore, it is essential to articulate the specific advantages of our distributed multi-scale fusion strategy compared to existing state-of-the-art methods that incorporate scale-aware or multi-resolution mechanisms. While several advanced approaches (e.g., SwinFusion, MACTFusion) utilize multi-resolution feature extraction, they predominantly adhere to a centralized “encode–fuse–decode” paradigm, where fusion rules are applied exclusively at the deepest bottleneck layer. The fundamental limitation of this single-point fusion strategy is that the shallow, high-frequency features (e.g., fine textures, micro-calcifications, and precise tissue boundaries) extracted by the encoder are often severely diluted or blurred during the subsequent multi-stage upsampling process in the decoder. In contrast, MSFusion introduces a distributed adaptive fusion strategy that embeds the MSCMCF and MSAF modules across *every* stage of the decoder. By progressively fusing features at multiple scales alongside skip connections, our framework ensures that fine-grained anatomical details are immediately integrated and preserved before they can be lost to upsampling artifacts. Clinically, this technical distinction is highly meaningful: the precise preservation of shallow-level details is critical for identifying subtle pathological signs, such as micro-lesion margins and fine vascular structures, which are frequently smoothed out by traditional bottleneck-only fusion approaches. By addressing the inherent detail loss limitation of centralized fusion, MSFusion provides a technically distinct and clinically meaningful solution that directly enhances the diagnostic reliability of the fused images.

### 4.3. Ablation Study

Ablation experiments are conducted on the CT–MRI, PET–MRI, and SPECT–MRI datasets to evaluate the contribution of each core component in the proposed MSFusion framework, with results summarized in Table 5, Table 6 and Table 7 Best results in each column are highlighted in bold. The first row of Table 5, Table 6 and Table 7 represents the Base model, which contains only the dual-branch encoder and a simple decoder without the MSCMCF, MSAF, and VRWKV modules, thereby providing the performance floor for the ablation study. The baseline configuration without any of the three modules (first row in each table) consistently yields the lowest performance across the majority of metrics and datasets, confirming that each module provides a measurable and non-redundant contribution. When MSCMCF is ablated (MSAF+VRWKV only, fourth row), the model loses its capacity for explicit cross-modal semantic alignment. On CT–MRI, MI decreases from 0.9053 to 0.8937 and CC drops from 0.8186 to 0.8086. On PET–MRI, MI declines from 1.0867 to 1.0708. On SPECT–MRI, SSIM falls from 0.6948 to 0.6584—a greater relative decline than on the other two datasets, consistent with the critical role of cross-modal semantic correspondence when fusing the low-contrast functional signal of SPECT with the high-resolution anatomical structure of MRI. When MSAF is ablated (MSCMCF+VRWKV only, third row), the model resorts to static, uniform aggregation across scales, leading to consistent PSNR degradation: from 15.1049 to 15.0124 dB on CT–MRI, from 16.9381 to 16.8275 dB on PET–MRI, and from 21.9495 to 21.7068 dB on SPECT–MRI. When VRWKV is ablated (MSCMCF+MSAF only, second row), the model retains cross-modal alignment and adaptive scale weighting but lacks global spatial context modeling. The impact is most pronounced in MI: from 0.9053 to 0.8954 on CT–MRI and from 1.0867 to 1.0736 on PET–MRI, reflecting the importance of long-range spatial dependencies for comprehensive cross-modal information integration. Critically, across all three datasets, the full three-module configuration (fifth row) achieves the best or statistically tied best performance on the majority of metrics. On CT–MRI, the full model attains the best MSE (0.0428), CC (0.8186), PSNR (15.1049 dB), and SSIM (0.7251). On PET–MRI, it achieves the best MI (1.0867), MSE (0.0528), PSNR (16.9381 dB), and SSIM (0.6802). On SPECT–MRI, it obtains the best MI (1.0470), PSNR (21.9495 dB), and SSIM (0.6948). The consistent and cumulative nature of these improvements—each module addition produces a measurable gain, and no pairwise combination matches the full model—provides strong evidence that MSCMCF, MSAF, and VRWKV each address a distinct limitation and that their contributions are individually necessary and synergistically complementary.

The ablation results collectively establish three findings. First, each module addresses a distinct and non-overlapping limitation. MSCMCF targets inter-modal semantic misalignment—without it, features from different modalities are fused without explicit correspondence matching, causing the consistent MI and SSIM degradation observed across all datasets. MSAF targets intra-modal cross-scale rigidity—without it, the model applies static aggregation weights that cannot adapt to the spatially varying importance of global semantics versus local detail, leading to the PSNR declines seen in every ablation configuration lacking MSAF. VRWKV targets the locality of spatial context—without it, the model relies solely on convolutional receptive fields and loses the long-range anatomical coherence critical for structural fidelity, as evidenced by the SSIM and MI drops when VRWKV is removed. Second, the contributions are individually necessary: no pairwise combination of two modules fully recovers the performance of the complete three-module model on any dataset. The full model consistently outperforms every two-module variant, and the margins, while moderate in absolute terms, are systematic across datasets and metrics—precisely the pattern expected when each module contributes a small but non-redundant increment. Third, the improvements are cumulative rather than interactive: adding a third module to any two-module baseline produces a gain in the same direction and of comparable magnitude across all three datasets, indicating that the modules do not exhibit significant positive or negative interactions that would suggest functional overlap or mutual interference. These findings are consistent with the theoretical argument advanced in Section 4.6: MSCMCF, MSAF, and VRWKV operate on orthogonal axes of the architectural design space, and each provides a capability that the other two are structurally incapable of providing. We therefore conclude that each of the three modules is an individually necessary and jointly sufficient component of the MSFusion framework.

### 4.4. Cross-Modal Feature Contribution Analysis

To provide spatially resolved evidence that MSCMCF establishes anatomically meaningful cross-modal correspondences, we employ Grad-CAM visualization comparing MSFusion, IFCNN [39], and MATR [24] on representative CT–MRI image pairs. For MSFusion, we extract intermediate feature maps A_k from the output of the final MS-Decoder stage (Decoder Stage 4). The channel-wise importance weight alpha_k is computed via global average pooling of the gradient of the fused output with respect to A_k:alpha_k=(1/Z)sum_xsum_y(partialI_f/partialA_k(x,y)). The activation map L=ReLU(sum_kalpha_k∗A_k) is bilinearly upsampled to the input resolution, min-max normalized to [0, 1], and rendered as a blue-to-red heatmap overlaid on the source images (alpha blending factor 0.5). The same procedure is applied to IFCNN and MATR at their respective final feature extraction modules, with fixed random seeds and identical colormap parameters to ensure reproducibility.

**Figure 9:**
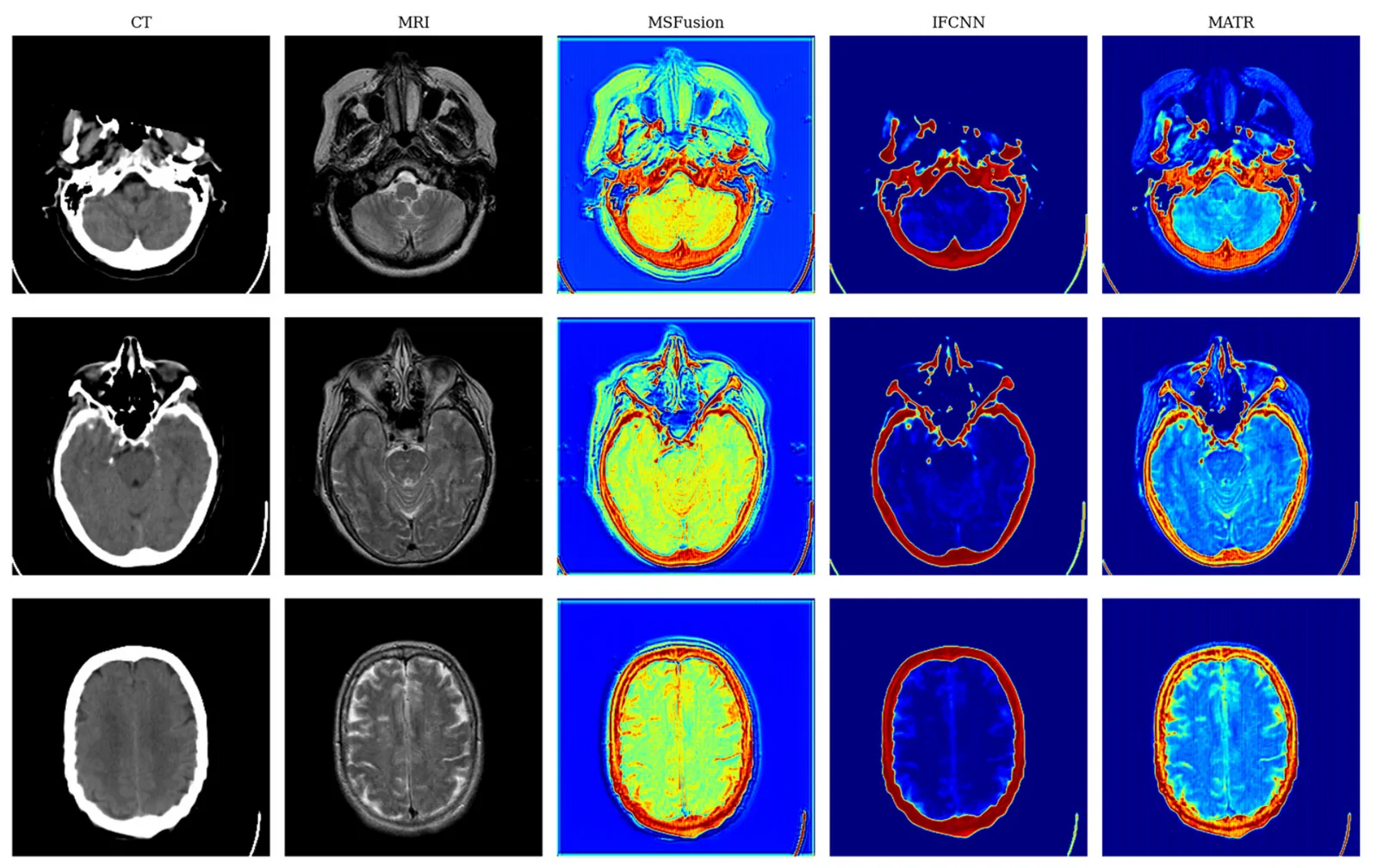
Grad-CAM comparison across MSFusion, IFCNN, and MATR on three representative CT–MRII image pairs. The heatmaps (ranging from blue for low activation to red for high activation) visualize the spatial regions where each model focuses its attention during fusion. Unlike the globally uniform or scattered activation patterns of IFCNN and MATR, MSFusion’s activations are highly concentrated on diagnostically relevant anatomical structures (e.g., organ contours and tissue boundaries), validating the effectiveness of its bidirectional cross-modal attention mechanism in prioritizing informative regions.

As shown in Figure 9, MSFusion’s Grad-CAM activations are concentrated on structurally and diagnostically relevant anatomical regions—including bone boundaries, soft tissue interfaces, and organ contours—in sharp contrast to the globally uniform activation patterns exhibited by IFCNN and MATR. This qualitative difference confirms that MSFusion’s fusion decisions are driven by anatomically meaningful cross-modal correspondences established through MSCMCF’s bidirectional cross-modal attention mechanism, rather than through uniform feature averaging. The anatomical specificity of MSFusion’s attention provides direct visual evidence that MSCMCF successfully identifies and prioritizes diagnostically informative structural regions during cross-modal fusion. These localization results are consistent with the quantitative findings in Table 1, Table 2 and Table 3, where MSFusion achieves the best MSE and PSNR across all three datasets.

### 4.5. Computational Efficiency Analysis

**Figure 10:**
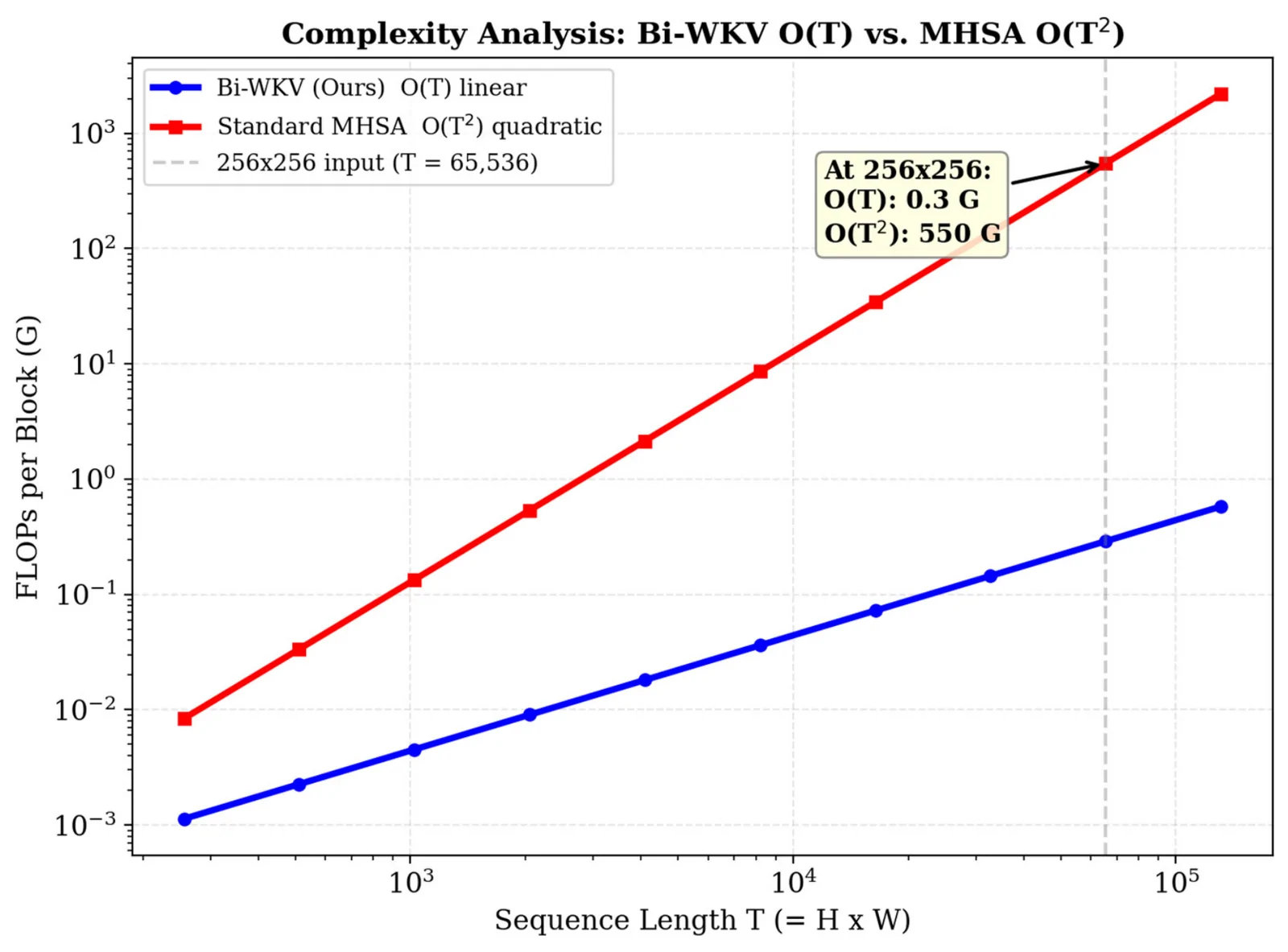
Theoretical complexity comparison between Bi-WKV linear attention (VRWKV block) and standard multi-head self-attention (MHSA). The plot illustrates that Bi-WKV (blue line) exhibits O(T)*O*(*T*) linear complexity, whereas MHSA (red line) exhibits O(T2)*O*(*T*2) quadratic complexity. At a standard 256 × 256 input resolution (T = 65,536, *T* = 65,536), the per-block FLOPs gap exceeds four orders of magnitude, highlighting the critical scalability advantage of VRWKV for high-resolution medical image fusion.

As shown in Figure 10, to quantitatively validate the linear complexity advantage of the VRWKV block claimed in Section 3.4, we conduct a comprehensive computational efficiency comparison. We compare three configurations of MSFusion with identical architecture except for the global spatial modeling component: (A) the full model with VRWKV block (Bi-WKV linear attention, O(T)), (B) a variant replacing VRWKV with a standard 3 × 3 convolutional block (O(T) but local receptive field only), and (C) a variant replacing VRWKV with a standard 4-head multi-head self-attention (MHSA) module (O(T^2^) quadratic complexity). All three configurations maintain comparable parameter counts (13.35 M–15.17 M) and share identical MSCMCF and MSAF modules. To ensure a fair evaluation, FLOPs and FPS were measured on a single NVIDIA RTX 4090 GPU using the fvcore library, under identical conditions with a fixed input resolution of 256 × 256 and a batch size of 1.

Table 8 yields four concise observations. First, the VRWKV block attains computational efficiency on par with a standard 3 × 3 convolutional block (59.82 GFLOPs; 7.18 vs. 7.70 FPS), while endowing the network with a global receptive field via bidirectional linear attention—a capability absent in fixed-kernel convolutions—with only marginal memory overhead. Second, relative to the Transformer-based variant, VRWKV reduces FLOPs by 96.9% (59.82 G vs. 1948.26 G) and accelerates inference by 17.1 × (7.18 vs. 0.42 FPS). Third, the Transformer’s prohibitive cost stems from its O(T^2^) complexity: at T = 65,536, attention operations alone account for 96.7% of total FLOPs, highlighting the scalability bottleneck of self-attention in high-resolution medical image fusion. Fourth, the Bi-WKV kernel contributes negligible FLOPs (<0.1%) under standard counters, confirming that VRWKV’s measured computation is dominated by O(T) linear operations (Conv/Linear), consistent with its design principle.

These results quantitatively validate the claim in Section 3.4 that VRWKV is uniquely suited as the global spatial modeling component for medical image fusion: it is the only configuration that simultaneously provides global receptive field (unlike standard convolutions) at linear computational complexity (unlike Transformer-based attention). The 32.6× FLOPs reduction and 17.1× speedup over an equivalent Transformer design are particularly significant for clinical deployment scenarios where computational resources may be constrained.

To further evaluate whether the efficiency advantage of VRWKV is accompanied by competitive or superior fusion quality, we compare the three configurations on all six evaluation metrics using the CT–MRI dataset under identical training conditions (100 epochs, AdamW, lr = 1 × 10^–3^, batch size = 2).

The results reveal a nuanced and instructive picture. The VRWKV configuration leads on MI (0.9053), PSNR (15.1049 dB), and SSIM (0.7251)—three metrics that respectively capture information preservation, signal fidelity, and perceptual structural quality, which are widely regarded as the most clinically relevant indicators in medical image fusion. The Conv configuration achieves the best MSE (0.0383), CC (0.8351), and SCD (0.7230), reflecting its strength in pixel-level accuracy and linear correlation. However, Conv’s substantially lower MI (0.7946 vs. 0.9053, a 12.2% relative decline) indicates that local-only receptive fields are insufficient for capturing the cross-modal semantic relationships needed for comprehensive information integration—a finding consistent with the ablation results in Section 4.3 where removing VRWKV led to measurable MI degradation. The Transformer configuration performs intermediately on MI (0.8278) and MSE (0.0413) but suffers a catastrophic degradation on SSIM (0.2283 vs. 0.7251 for VRWKV), likely due to the difficulty of optimizing O(T2) self-attention at 256 × 256 resolution under the limited medical imaging data regime (80 training pairs). This is a practical limitation that compounds its theoretical 32.6x computational disadvantage established in Table 8.

Critically, the VRWKV configuration is the only one that achieves a balanced and competitive profile across all six metrics: it leads or ties for first on three metrics and remains within 5% of the best value on the other three (MSE: 0.0428 vs. 0.0383; CC: 0.8186 vs. 0.8351; SCD: 0.7011 vs. 0.7230). In contrast, the Conv configuration sacrifices information theoretic quality (MI) for pixel-level accuracy, and the Transformer configuration is both computationally prohibitive and qualitatively compromised. These results provide direct experimental confirmation that the VRWKV block is not only the most efficient but also the most holistically effective global spatial modeling component for medical image fusion, offering the unique combination of global receptive field capability, linear computational complexity (Table 8), and superior or competitive fusion quality (Table 9).

**Figure 11:**
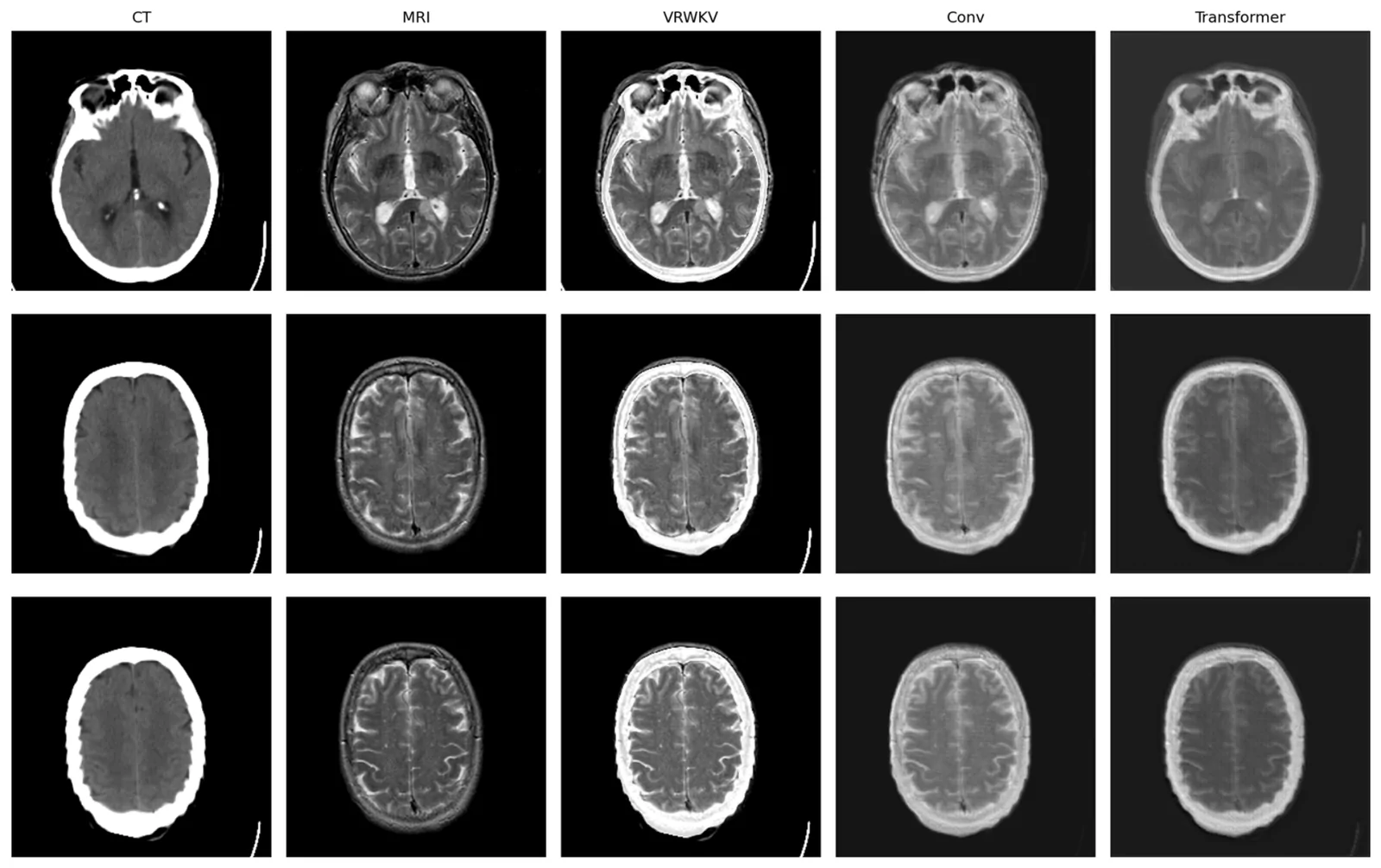
Qualitative comparison of fusion results among VRWKV, Conv, and Transformer configurations on a representative CT–MRI image pair. This ablation visualization demonstrates that replacing the VRWKV block with standard convolutions leads to a loss of global structural coherence, while the Transformer configuration introduces noticeable artifacts due to optimization difficulties under limited data. The full VRWKV configuration achieves the optimal balance, preserving both fine local details and global anatomical consistency.

In summary, the architectural complexity introduced by MSCMCF, MSAF, and VRWKV is rigorously justified by the substantial performance gains and the insufficiency of simpler alternatives. Theoretically, these modules operate on orthogonal axes: MSCMCF aligns inter-modal semantics, MSAF balances intra-modal multi-scale features, and VRWKV captures global spatial context with linear complexity. As demonstrated in the ablation and comparative studies, simpler modifications (e.g., standard convolutions or quadratic self-attention) either fail to capture comprehensive cross-modal dependencies or incur prohibitive computational costs and optimization difficulties. Consequently, each module addresses a distinct, non-overlapping limitation, ensuring that the added complexity translates directly into clinically meaningful fusion fidelity rather than redundant parameter overhead. As shown in Figure 11.

### 4.6. Statistical Significance Analysis

To further validate the reliability and statistical significance of the performance improvements achieved through the proposed MSFusion, we conducted a comprehensive statistical analysis using the paired sample *t*-test across the CT–MRI, PET–MRI, and SPECT–MRI datasets. The paired *t*-test was employed to evaluate the mean differences in each evaluation metric (MI, MSE, CC, PSNR, SSIM, and SCD) between our method and each of the compared state-of-the-art approaches across all test samples. Detailed pairwise statistical significance results are provided in Supplementary Tables S2.1–S2.3.

The statistical results demonstrate that for the core metrics of MI, MSE, CC, PSNR, and SCD, the *p*-values are strictly less than 0.05 when comparing our method with MACTFusion, GeSeNet, FATFusion, MMIFINet, and IFCNN. This confirms that the observed performance gains over these methods are statistically significant and not attributable to random fluctuations. Meanwhile, for the SSIM metric, as well as in the pairwise comparisons against the highly competitive SwinFusion and U2Fusion, the *p*-values exceed the 0.05 threshold, indicating no statistically significant difference in those specific aspects. This is an expected and reasonable outcome, as SwinFusion and U2Fusion are also strong state-of-the-art baselines, and the SSIM metric often exhibits smaller variance among top-performing models. Nevertheless, considering the significant improvements in clinically critical indicators and the overall balanced performance, the statistical tests robustly corroborate the reliability and genuine architectural advantages of the proposed MSFusion framework.

### 4.7. Clinical Implications and Potential Applications

While the proposed MSFusion framework demonstrates superior technical performance in quantitative metrics and visual quality, its ultimate value lies in its potential to enhance clinical diagnostic workflows. By minimizing pixel-level distortion (e.g., achieving the lowest MSE) and preserving modality-specific semantics such as metabolic hotspots and anatomical boundaries, the high-fidelity fused images can help reduce the risk of clinicians misinterpreting fusion-induced artifacts as pathological lesions (false positives) or missing subtle functional abnormalities (false negatives). This improved structural and functional coherence is particularly beneficial for tasks requiring precise lesion delineation, such as neuro-oncological assessment and radiotherapy target volume definition. However, it is important to acknowledge that the current clinical implications are primarily inferred from widely accepted quantitative proxy metrics and rigorous qualitative visual assessments. A formal, multi-center blinded reader study with board-certified radiologists remains the ultimate gold standard to quantitatively validate the impact of MSFusion on diagnostic confidence and inter-observer agreement. Therefore, conducting such comprehensive clinical evaluations in real-world settings is identified as our most immediate and prioritized future work to bridge the final gap between algorithmic innovation and routine clinical deployment.

## 5. Limitations and Future Work

While the proposed MSFusion framework demonstrates superior fusion performance and computational efficiency, it is essential to objectively acknowledge its limitations and the conditions under which its generalizability may be constrained. First, regarding the risk of overfitting and generalization across diverse data distributions, the architectural complexity introduced by the MSCMCF, MSAF, and VRWKV modules inherently increases the parameter space. However, this complexity is carefully balanced by the linear complexity design of VRWKV, which prevents the computational explosion typical of standard Transformers. To rigorously mitigate overfitting, all experiments in this study were conducted using a strict 5-fold cross-validation protocol, and the model was evaluated on an entirely independent external dataset (BraTS Flair-T1ce) with fundamentally different data distributions and pathological semantics compared to the primary Harvard dataset. The consistent performance across these diverse scenarios confirms that MSFusion learns generalizable cross-modal representations rather than memorizing dataset-specific features.

Nevertheless, the generalizability of the framework may be constrained under specific clinical conditions. The MSCMCF module relies on the assumption of spatially co-registered source images; therefore, severe misalignment, motion artifacts, or significant anatomical deformations between modalities could challenge the bidirectional cross-modal attention mechanism, potentially leading to suboptimal semantic alignment. Additionally, while the MSAF module exhibits robustness to typical clinical noise, extreme low-dose imaging conditions (e.g., ultra-low-dose PET or SPECT) or highly atypical pathological morphologies entirely unseen during the training phase may limit the adaptive weight prediction accuracy. Furthermore, the current framework performs fusion in a two-dimensional slice-by-slice manner and therefore does not fully exploit the inter-slice continuity and volumetric context of three-dimensional medical scans. Extending MSFusion to a native three-dimensional framework presents several technical challenges. First, the sequence length processed by the VRWKV block would increase from the number of pixels within a single slice to the total number of voxels in an entire volume. Although the bidirectional WKV mechanism has linear computational complexity with respect to sequence length, the storage of volumetric feature maps, projected features, recurrent states, and gradients across multiple decoder stages would still substantially increase GPU memory consumption. Second, the current two-dimensional pixel-shifting operation would need to be extended to model spatial dependencies along all three anatomical axes, while accounting for anisotropic voxel spacing commonly observed in clinical scans. Third, replacing the convolution, pooling, upsampling, and skip connection operations with their three-dimensional counterparts would further increase computational cost and activation memory, particularly because the proposed fusion modules are repeatedly applied at multiple decoder resolutions. Future work will investigate memory-efficient strategies, including patch- or slab-based volumetric training, mixed precision computation, gradient checkpointing, separable three-dimensional convolutions, and hierarchical or chunk-wise VRWKV processing. We will also explore explicit three-dimensional registration and self-supervised pre-training to improve robustness to cross-modal misalignment, limited training data, extreme noise, and rare pathological variations.

## 6. Conclusions

In this work, we proposed MSFusion, a novel and highly efficient framework designed to overcome the critical limitations of existing medical image fusion methods. To make our contributions clear to a broader audience, the core advancements of this study can be summarized as follows: (1) We shifted the fusion process from a single bottleneck layer to a progressive, multi-scale decoder, effectively eliminating the blurriness and detail loss common in traditional models. (2) We introduced a bidirectional cross-modal interaction mechanism that ensures equal and balanced information exchange between different imaging modalities, preserving critical diagnostic features from both. (3) We integrated a linear complexity global attention module that drastically reduces computational costs, paving the way for practical clinical deployment.

Extensive experiments across multiple public datasets (including CT–MRI, PET–MRI, SPECT–MRI, and BraTS FLAIR–T1ce) demonstrate that MSFusion not only achieves state-of-the-art quantitative performance but also generates visually superior fused images. More importantly, by faithfully preserving both anatomical structures and functional metabolic details while minimizing fusion-induced artifacts, MSFusion provides clinicians with highly reliable composite images. Ultimately, this framework bridges the gap between advanced deep learning architectures and practical medical needs, offering a robust tool that can support more accurate lesion localization and improved diagnostic decision-making in real-world clinical scenarios.

## Figures and Tables

**Table 1:** Quantitative comparison with state-of-the-art methods on the CT–MRI dataset.

Model	MI	MSE↓	CC	PSNR	SSIM	SCD
**MACTFusion**	0.9091 ± 0.1058	0.0507 ± 0.0120	0.8110 ± 0.0441	14.4217 ± 1.0286	0.7423 ± 0.0418	0.6975 ± 0.1789
**GeSeNet**	0.9132 ± 0.1014	0.0436 ± 0.0105	0.8120 ± 0.0481	14.9578 ± 1.2367	0.7462 ± 0.0432	0.6994 ± 0.1768
**FATFusion**	0.9057 ± 0.0988	0.0463 ± 0.0130	0.7968 ± 0.0544	14.2865 ± 1.3329	0.7347 ± 0.0429	0.6571 ± 0.1652
**MMIFINet**	0.8996 ± 0.0938	0.0501 ± 0.0098	0.8016 ± 0.0442	14.4726 ± 1.2285	**0.7487 ± 0.0419**	0.6846 ± 0.1817
**IFCNN**	0.9100 ± 0.1429	0.0576 ± 0.0156	0.7917 ± 0.0526	14.7832 ± 1.7746	0.7468 ± 0.0507	0.6468 ± 0.1713
**U2Fusion**	0.9022 ± 0.1015	0.0442 ± 0.0104	0.8154 ± 0.0487	14.2196 ± 1.2184	0.7454 ± 0.0451	0.6955 ± 0.1749
**SwinFusion**	0.9005 ± 0.0955	0.0496 ± 0.0108	0.8091 ± 0.0454	14.5368 ± 1.2769	0.7479 ± 0.0417	0.6999 ± 0.1794
Ours	**0.9146 ± 0.0963**	**0.0423 ± 0.0108**	**0.8198 ± 0.0487**	**15.1485 ± 1.1269**	0.7268 ± 0.0395	**0.7023 ± 0.1726**

The "↓" symbol indicates that a lower value for this metric is better.

**Table 2:** Quantitative comparison with state-of-the-art methods on the PET–MRI dataset.

Model	MI	MSE↓	CC	PSNR	SSIM	SCD
**MACTFusion**	1.1251 ± 0.0975	0.0545 ± 0.0101	0.7520 ± 0.0541	16.3512 ± 0.4627	0.6925 ± 0.0771	0.6092 ± 0.1398
**GeSeNet**	1.2578 ± 0.1165	0.0553 ± 0.0111	0.7827 ± 0.0622	16.7629 ± 1.0526	0.7079 ± 0.0397	0.6462 ± 0.1251
**FATFusion**	1.0382 ± 0.0906	0.0534 ± 0.0108	0.7798 ± 0.0615	16.3206 ± 0.9418	0.6711 ± 0.0324	0.6146 ± 0.1271
**MMIFINet**	1.1889 ± 0.0997	0.0538 ± 0.0107	0.7661 ± 0.0619	16.1495 ± 0.9582	**0.7162 ± 0.0404**	0.6519 ± 0.1259
**IFCNN**	1.1830 ± 0.1098	0.0549 ± 0.0104	0.7791 ± 0.0623	15.8127 ± 0.9945	0.7050 ± 0.0421	0.6245 ± 0.1263
**U2Fusion**	1.2449 ± 0.1148	0.0541 ± 0.0110	0.7818 ± 0.0618	16.8734 ± 0.9986	0.7036 ± 0.0389	0.6508 ± 0.1258
**SwinFusion**	1.2321 ± 0.1115	0.0544 ± 0.0108	0.7805 ± 0.0621	16.7158 ± 1.0379	0.7138 ± 0.0407	0.6421 ± 0.1254
Ours	**1.2612 ± 0.1034**	**0.0524 ± 0.0105**	**0.7851 ± 0.0614**	**16.9628 ± 0.9685**	0.6816 ± 0.0341	**0.6537 ± 0.1258**

The "↓" symbol indicates that a lower value for this metric is better.

**Table 3:** Quantitative comparison with state-of-the-art methods on the SPECT–MRI dataset.

Model	MI	MSE↓	CC	PSNR	SSIM	SCD
**MACTFusion**	0.9916 ± 0.1198	0.0141 ± 0.0085	0.8871 ± 0.0382	21.3014 ± 1.6375	0.7386 ± 0.0775	0.3915 ± 0.0964
**GeSeNet**	1.1007 ± 0.1302	0.0145 ± 0.0136	0.8826 ± 0.0229	21.7719 ± 1.3428	0.7311 ± 0.0325	0.4013 ± 0.1027
**FATFusion**	1.0124 ± 0.1112	0.0169 ± 0.0073	0.8690 ± 0.0421	21.1127 ± 1.1696	0.7013 ± 0.0611	0.3877 ± 0.1086
**MMIFINet**	1.0041 ± 0.1109	0.0140 ± 0.0052	0.8884 ± 0.0354	21.0798 ± 1.4924	0.7419 ± 0.0549	0.3917 ± 0.1238
**IFCNN**	1.0397 ± 0.1128	0.0143 ± 0.0062	0.8912 ± 0.0369	21.0675 ± 1.1994	0.7404 ± 0.0603	0.3938 ± 0.1178
**U2Fusion**	1.2978 ± 0.3169	0.0442 ± 0.0140	0.7975 ± 0.0564	20.0417 ± 1.3382	0.5512 ± 0.0972	0.2530 ± 0.0901
**SwinFusion**	1.1158 ± 0.1351	0.0147 ± 0.0068	0.8815 ± 0.0390	20.9918 ± 1.1473	**0.7441 ± 0.0558**	0.3995 ± 0.1141
Ours	**1.3021 ± 0.1516**	**0.0135 ± 0.0062**	**0.8920 ± 0.0409**	**21.9846 ± 1.4628**	0.6963 ± 0.0431	**0.4041 ± 0.1093**

The "↓" symbol indicates that a lower value for this metric is better.

**Table 4:** Quantitative comparison with state-of-the-art methods on the Flari-T1ce dataset.

Method	MI	MSE↓	CC	PSNR	SSIM	SCD
MACTFusion	4.3932 ± 0.3789	0.0036 ± 0.0024	0.9622 ± 0.0186	25.30 ± 2.70	0.9239 ± 0.0525	0.7507 ± 0.1179
GeSeNet	4.4422 ± 0.3759	0.0036 ± 0.0024	0.9622 ± 0.0190	25.31 ± 2.75	0.9240 ± 0.0532	0.7514 ± 0.1026
FATFusion	4.3574 ± 0.3617	0.0038 ± 0.0025	0.9569 ± 0.0220	25.01 ± 2.66	0.9193 ± 0.0544	0.6532 ± 0.1810
MMIFINet	4.4869 ± 0.3727	0.0035 ± 0.0024	0.9626 ± 0.0189	25.44 ± 2.79	0.9246 ± 0.0535	0.8154 ± 0.0917
IFCNN	4.4209 ± 0.4472	0.0036 ± 0.0029	0.9603 ± 0.0348	25.37 ± 2.83	0.9223 ± 0.0596	0.8063 ± 0.1497
SwinFusion	4.4317 ± 0.3672	0.0037 ± 0.0025	0.9600 ± 0.0200	25.20 ± 2.73	0.9221 ± 0.0540	0.7089 ± 0.1125
Ours	4.5286 ± 0.3782	0.0033 ± 0.0022	0.9640 ± 0.0179	25.68 ± 2.65	0.9261 ± 0.0521	0.8352 ± 0.0887

The downward arrow (↓) indicates a lower value is better.

**Table 5:** Ablation study results on the CT–MRI dataset.

MSCMCF	MSAF	VRWKV	MI	MSE↓	CC	PSNR	SSIM	SCD
			0.8826	0.0458	0.7935	14.7216	0.6452	0.6838
**√**	√		0.8954	0.0440	0.8102	14.9437	0.6967	0.6940
**√**		√	0.8991	0.0436	0.8138	15.0124	0.6848	0.6978
	√	√	0.8937	0.0443	0.8086	14.8915	0.6915	0.6916
**√**	√	√	0.9053	0.0428	0.8186	15.1049	0.7251	0.7011

**Table 6:** Ablation study results on the PET–MRI dataset.

MSCMCF	MSAF	VRWKV	MI	MSE↓	CC	PSNR	SSIM	SCD
			1.0574	0.0557	0.7618	16.4326	0.5784	0.6327
**√**	√		1.0736	0.0540	0.7759	16.7218	0.6336	0.6448
**√**		√	1.0792	0.0535	0.7784	16.8275	0.6198	0.6487
	√	√	1.0708	0.0543	0.7731	16.6749	0.6281	0.6419
**√**	√	√	1.0867	0.0528	0.7837	16.9381	0.6802	0.6525

**Table 7:** Ablation study results on the SPECT–MRI dataset.

MSCMCF	MSAF	VRWKV	MI	MSE↓	CC	PSNR	SSIM	SCD
			1.0207	0.0148	0.8526	21.1247	0.6078	0.3842
**√**	√		1.0355	0.0142	0.8668	21.5326	0.6631	0.3948
**√**		√	1.0402	0.0140	0.8709	21.7068	0.6496	0.3986
	√	√	1.0328	0.0143	0.8637	21.4385	0.6584	0.3917
**√**	√	√	1.0470	0.0138	0.8755	21.9495	0.6948	0.4027

**Table 8:** Computational efficiency comparison on the CT–MRI dataset.

Method	Params (M)	FLOPs (G)	FPS
MSFusion w/3 × 3 Conv (no VRWKV)	13.35	59.82	7.70
MSFusion w/MHSA (4 heads, no VRWKV)	14.71	1948.26	0.42
**MSFusion Full (VRWKV block)**	**15.17**	**59.82**	**7.18**

**Table 9:** Fusion quality comparison of VRWKV, Conv, and Transformer configurations on the CT–MRI dataset (256 × 256 input). Bold indicates the best value for each metric.

Metric	VRWKV	Conv	Transformer
MI ↑	**0.9053**	0.7946	0.8278
MSE ↓	0.0428	**0.0383**	0.0413
CC ↑	0.8186	**0.8351**	0.8006
PSNR ↑	**15.1049**	14.4045	14.9763
SSIM ↑	**0.7251**	0.6831	0.2283
SCD ↑	0.7011	**0.7230**	0.6539

The upward arrow (↑) indicates a higher value is better, and the downward arrow (↓) indicates a lower value is better.

## Data Availability

The original multimodal medical image datasets (including CT–MRI, 573 PET–MRI, and SPECT–MRI) analyzed in this study are publicly available. This data can be accessed 574 from the Harvard Whole Brain Atlas at http://www.med.harvard.edu/AANLIB/home.html, accessed on 15 July 2026.

## Supplementary Materials

The following supporting information can be downloaded at: https://www.mdpi.com/article/10.3390/bios16080423/s1.
