# Supplementary material for: MSFusion: Multi-Scale Cross-Modal Fusion with Adaptive Attention for Multimodal Medical Image Fusion
Source: Biosensors (Basel). 2026 Aug 6;16(8):423. doi: 10.3390/bios16080423 (PMC13510988; doi:10.3390/bios16080423)
Supplement: Supplementary file 1 [file biosensors-16-00423-s001.zip › biosensors-4384289-supplementary.pdf]

Supplementary Material

S1. Notation Table

This section provides a comprehensive notation table that defines all mathematical symbols, abbreviations, and terminology used in the main manuscript. The notation is organized by module to facilitate understanding of the proposed methodology.

S1.1 General Symbols

Symbol	Definition
I_A, I_B	Input multimodal medical images (e.g., CT-MRI, PET-MRI, SPECT-MRI pairs)
$I_f$or I	Fused image preserving complementary structural and functional information
B	Batch size
C	Number of channels
H, W	Feature map height and width
T	Sequence length ($T = H$ x W)
Concat	Concatenation operation along channel dimension

S1.2 Multi-Scale Cross-Modal Cooperative Fusion (MSCMCF) Module The MSCMCF module establishes deep semantic correspondence between modalities through a bidirectional cross-modal attention mechanism.

Symbol	Definition
MSCMCF	Multi-Scale Cross-Modal Cooperative Fusion module
Q_A, Q_B	Query features for modality A and B, derived from input features
K_A, K_B	Key features for modality A and B, used for attention computation
V_A, V_B	Value features for modality A and B, used for feature extraction
SA	Spatial Attention: identifies spatially co-localized features
CA	Channel Attention: identifies semantically relevant feature channels
sigma	Sigmoid activation function: sigma(x) = 1/(1 + exp(-x))
F_A^g, F_B^g	Global-scale features for modality A and B
F_A^l, F_B^l	Local-scale features for modality A and B
ConvBlock	Basic convolutional block: Conv + BatchNorm + ReLU
W_A, W_B	Learnable enhancement weights for modality A and B
DWC(k)	Depthwise Separable Convolution with kernel size k
w	Learnable spatial decay parameter for cross-modal attention
beta	Learnable residual weight parameter for training stability
F_{CMCF}	Output features from MSCMCF module

S1.3 Multi-Scale Adaptive Fusion (MSAF) Module The MSAF module dynamically assigns fusion weights across scales to balance global semantics and local details.

Symbol	Definition
MSAF	Multi-Scale Adaptive Fusion module
GAP	Global Average Pooling operation
w^g	Adaptive fusion weight for global-scale features
w^l	Adaptive fusion weight for local-scale features
F_{fuse}	Multi-scale fused features output
alpha	Learnable residual weight for detail preservation
F_{MSAF}	Output features from MSAF module

S1.4 Vision RWKV (VRWKV) Block The VRWKV block enables efficient global spatial feature modeling with linear computational complexity.

Symbol	Definition
VRWKV	Vision RWKV Block for spatial feature mixing
Bi-WKV	Bidirectional WKV: linear-complexity attention mechanism
Shift(s)	Channel-wise pixel shifting with shift ratio s
R	Receptance vector for gating mechanism
o_t	Attention output at spatial position t
u	Bonus parameter: ensures current position not suppressed by decay
s_t	Cumulative spatial state at position t
exp	Exponential function
Sigmoid	Sigmoid activation: sigma(x) = 1/(1 + exp(-x))
W_o	Output projection matrix
F_{VRWKV}	Output features from VRWKV module

S1.5 Evaluation Metrics

Symbol	Full Name	Description
MSE	Mean Squared Error	Pixel-level accuracy metric; lower indicates better fusion quality
PSNR	Peak Signal-to-Noise Ratio	Signal fidelity metric in dB; higher indicates better quality
SSIM	Structural Similarity Index	Perceptual quality metric [0,1]; higher indicates better structure preservation
MI	Mutual Information	Information preservation metric; higher indicates more shared information
CC	Cross-Correlation	Linear correlation metric [-1,1]; higher indicates stronger correlation

SCD	Structural Contrast Difference	Contrast preservation metric; higher indicates better contrast
EN	Entropy	Information content metric; higher indicates more information
SF	Spatial Frequency	Detail preservation metric; higher indicates more details
AG	Average Gradient	Edge sharpness metric; higher indicates sharper edges

S1.6 Loss Functions

**Symbol**	**Definition**
L_{total}	Total loss function: weighted combination of all loss terms
L_{pixel}	Pixel-level reconstruction loss
$L_{perceptual}$	Perceptual loss based on pretrained network features
$L_{gradient}$	Gradient loss for edge preservation
L_{SSIM}	SSIM-based loss for structural similarity maximization

S1.7 Network Architecture

**Symbol**	**Definition**
E_A, E_B	Dual-branch convolutional encoders for modality A and B
MS-Decoder	Multi-level decoder module cascade
Stage i	i-th decoder stage (i = 1, 2, 3, 4)
ConvBlock	Sequentially stacked convolutional blocks
Skip-Connection	Skip connections for multi-scale feature integration
VRWKV	Vision RWKV blocks inserted after each MS-Decoder

S1.8 Complexity Notation

**Symbol**	**Definition**
O(T)	Linear complexity with respect to sequence length T
$O(T^2)$	Quadratic complexity with respect to sequence length T
O(Td)	Linear complexity with feature dimensionality d
O(d log T)	Parallel computation depth
GFLOPs	Giga Floating Point Operations Per Second
FPS	Frames Per Second (inference speed)

S2. Statistical Significance Testing

Tables S2–S4 summarize the statistical significance of the pairwise comparisons between MSFusion and the competing methods on the CT–MRI, PET–MRI, and SPECT–MRI datasets, respectively. A value of $p < 0.05$ indicates a statistically significant difference, whereas $p > 0.05$ indicates that the difference is not statistically significant.

Table S2.1. Statistical significance of pairwise comparisons on the CT–MRI dataset

Compared Method	MI	MSE	CC	PSNR	SSIM	SCD
MACTFusion	$p < 0.05$	$p < 0.05$	$p < 0.05$	$p < 0.05$	$p > 0.05$	$p < 0.05$
GeSeNet	$p < 0.05$	$p < 0.05$	$p < 0.05$	$p < 0.05$	$p > 0.05$	$p < 0.05$
FATFusion	$p < 0.05$	$p < 0.05$	$p < 0.05$	$p < 0.05$	$p > 0.05$	$p < 0.05$
MMIFINet	$p < 0.05$	$p < 0.05$	$p < 0.05$	$p < 0.05$	$p > 0.05$	$p < 0.05$
IFCNN	$p < 0.05$	$p < 0.05$	$p < 0.05$	$p < 0.05$	$p > 0.05$	$p < 0.05$
U2Fusion	$p > 0.05$	$p > 0.05$	$p > 0.05$	$p > 0.05$	$p > 0.05$	$p > 0.05$
SwinFusion	$p > 0.05$	$p > 0.05$	$p > 0.05$	$p > 0.05$	$p > 0.05$	$p > 0.05$

Table S2.2. Statistical significance of pairwise comparisons on the PET–MRI dataset

Compared Method	MI	MSE	CC	PSNR	SSIM	SCD
MACTFusion	$p < 0.05$	$p < 0.05$	$p < 0.05$	$p < 0.05$	$p > 0.05$	$p < 0.05$
GeSeNet	$p < 0.05$	$p < 0.05$	$p < 0.05$	$p < 0.05$	$p > 0.05$	$p < 0.05$
FATFusion	$p < 0.05$	$p < 0.05$	$p < 0.05$	$p < 0.05$	$p > 0.05$	$p < 0.05$
MMIFINet	$p < 0.05$	$p < 0.05$	$p < 0.05$	$p < 0.05$	$p > 0.05$	$p < 0.05$
IFCNN	$p < 0.05$	$p < 0.05$	$p < 0.05$	$p < 0.05$	$p > 0.05$	$p < 0.05$
U2Fusion	$p > 0.05$	$p > 0.05$	$p > 0.05$	$p > 0.05$	$p > 0.05$	$p > 0.05$
SwinFusion	$p > 0.05$	$p > 0.05$	$p > 0.05$	$p > 0.05$	$p > 0.05$	$p > 0.05$

Table S2.3. Statistical significance of pairwise comparisons on the SPECT–MRI dataset

Compared Method	MI	MSE	CC	PSNR	SSIM	SCD
MACTFusion	$p < 0.05$	$p < 0.05$	$p < 0.05$	$p < 0.05$	$p > 0.05$	$p < 0.05$
GeSeNet	$p < 0.05$	$p < 0.05$	$p < 0.05$	$p < 0.05$	$p > 0.05$	$p < 0.05$
FATFusion	$p < 0.05$	$p < 0.05$	$p < 0.05$	$p < 0.05$	$p > 0.05$	$p < 0.05$
MMIFINet	$p < 0.05$	$p < 0.05$	$p < 0.05$	$p < 0.05$	$p > 0.05$	$p < 0.05$
IFCNN	$p < 0.05$	$p < 0.05$	$p < 0.05$	$p < 0.05$	$p > 0.05$	$p < 0.05$
U2Fusion	$p > 0.05$	$p > 0.05$	$p > 0.05$	$p > 0.05$	$p > 0.05$	$p > 0.05$
SwinFusion	$p > 0.05$	$p > 0.05$	$p > 0.05$	$p > 0.05$	$p > 0.05$	$p > 0.05$
